# Biological insights from multi-omics analysis strategies: Complex pleotropic effects associated with autophagy

**DOI:** 10.3389/fpls.2023.1093358

**Published:** 2023-02-16

**Authors:** Geng Ding, Yosia Mugume, Maria Emilia Dueñas, Young Jin Lee, Meiling Liu, Daniel S. Nettleton, Xuefeng Zhao, Ling Li, Diane C. Bassham, Basil J. Nikolau

**Affiliations:** ^1^ Roy J. Carver Department of Biochemistry, Biophysics and Molecular Biology, Iowa State University, Ames, IA, United States; ^2^ Department of Genetics, Development and Cell Biology, Iowa State University, Ames, IA, United States; ^3^ Department of Chemistry, Iowa State University, Ames, IA, United States; ^4^ Department of Statistics, Iowa State University, Ames, IA, United States; ^5^ Research Information Technology, College of Liberal Arts & Sciences, Iowa State University, Ames, IA, United States; ^6^ Department of Biological Sciences, Mississippi State University, Mississippi State, MS, United States

**Keywords:** autophagy, multi-omics, lipidomics, RNA-seq, arabidopsis

## Abstract

Research strategies that combine molecular data from multiple levels of genome expression (i.e., multi-omics data), often referred to as a systems biology strategy, has been advocated as a route to discovering gene functions. In this study we conducted an evaluation of this strategy by combining lipidomics, metabolite mass-spectral imaging and transcriptomics data from leaves and roots in response to mutations in two *AuTophaGy-related* (*ATG*) genes of *Arabidopsis*. Autophagy is an essential cellular process that degrades and recycles macromolecules and organelles, and this process is blocked in the *atg7* and *atg9* mutants that were the focus of this study. Specifically, we quantified abundances of ~100 lipids and imaged the cellular locations of ~15 lipid molecular species and the relative abundance of ~26,000 transcripts from leaf and root tissues of WT, *atg7* and *atg9* mutant plants, grown either in normal (nitrogen-replete) and autophagy-inducing conditions (nitrogen-deficient). The multi-omics data enabled detailed molecular depiction of the effect of each mutation, and a comprehensive physiological model to explain the consequence of these genetic and environmental changes in autophagy is greatly facilitated by the *a priori* knowledge of the exact biochemical function of the ATG7 and ATG9 proteins.

## Introduction

Beginning with the investments that supported the completion of the Human Genome Project (www.genome.gov/human-genome-project), many analytical advances have enabled the rapid expansion in the number of genomes that are now fully sequenced. Thus, at the time of writing (July, 2022) NCBI had cataloged over 70,000 annotated genomes (https://www.ncbi.nlm.nih.gov/genome/browse#!/overview), and this number is rapidly expanding. For example, the Earth BioGenome Project (www.earthbiogenome.org) envisions sequencing the genomes of all known eukaryotic organisms; approximately 1.8 million species.

With the prospect of having access to such an enormous data resource, namely the genetic blueprint of a large portion of the earth’s eukaryotic biosphere, one can ask the question, are we, the scientific endeavor, prepared to take advantage of this opportunity to better understand the complexity of biology. Although there are many ways that one can address this question, at the core is the ability to identify the structure and function of individual genes as they integrate in a system that maintains biological veracity and viability. In this context, since 2000 the plant biology research community has had access to high quality genome sequence for *Arabidopsis thaliana* (L.) Heynh (Arabidopsis Genome, 2000; M. et al., 2001), and this has empowered many advances in the plant sciences. Indeed, with research support from the National Science Foundation, the Arabidopsis 2010 projects (www.arabidopsis.org/portals/masc/projects.jsp) led to increased definitions of gene functions, as captured in The Arabidopsis Information Resource database (www.arabidopsis.org) (S. Y. et al., 2003; Reiser et al., 2017), and this progress has been cataloged in the annual reports from the Multinational Arabidopsis Steering Committee (Parry et al., 2020). Analogous resources are now being assembled for other plant species, with obvious practical expansion to the major crops (e.g., maize, rice, wheat etc.). Yet despite these advances, approximately 30% of the annotated genes in most sequenced genomes are still annotated as either “hypothetical genes” or “genes of unknown function”, and even for those genes that are annotated with some functionality, these are often with ambiguous annotations (e.g., Lobb et al., 2020). In addition, more recent genomic characterizations have identified the potential of “orphan genes” to determine and regulate novel traits (Arendsee et al., 2014).

Overcoming this challenge with new sophisticated instrumentation has empowered the measurement of numerous molecular attributes of biological systems in a high throughput manner, which has enabled the global expression profile of the genome at the level of the transcriptome, proteome and metabolome. Collecting these comprehensive datasets are often justified as resources that will provide insights to gene-function. Yet, despite this enhanced ability to generate multi-platform data, it’s not obvious how these data inputs have enhanced the ability to discover new gene-functions. In this study we evaluated this question by applying integrated transcriptomics and metabolomics strategies on two *Arabidopsis* mutants in genes whose biological function is known by the fact that these mutations affect the process of autophagy, and *in vitro* characterizations have provided accurate insights on the biochemical functions of each gene-product.

Autophagy is a degradation pathway that engulfs organelles and macromolecules into vesicles, called autophagosomes and thereby transfers them to the vacuole, where they are degraded, and breakdown products are recycled (Yu et al., 2018). Genetic dissection of autophagy has identified a collection of mutants that affect this process, and these are collectively known as *AuTophaGy-related* (*ATG*) genes. These were initially characterized in the yeast, *Saccharomyces cerevisiae* (Tsukada and Ohsumi, 1993; Farre and Subramani, 2016; Majeed et al., 2022), and homologs have also been characterized from a wide range of eukaryotes (Yin et al., 2016), including plants (Soto-Burgos et al., 2018). For the purpose of this study, we selected *Arabidopsis* mutants in two of these genes, *ATG9* (AT2G31260) and *ATG7* (AT5G45900), whose biochemical functions have been identified by extensive characterizations, particularly in yeast and humans. Moreover, the specific function of ATG9 in plants is based on structural conservation of orthologs from *Arabidopsis* (Lai et al., 2020), yeast (Matoba et al., 2020) and humans (Guardia et al., 2020), which speaks to the phylogenetic conservation of the autophagic process. Thus, ATG9 is a lipid scramblase (Matoba et al., 2020) and ATG7 is an E1-like enzyme that catalyzes the covalent conjugation of the ATG12 and ATG5 proteins (Mizushima et al., 1998) and covalent attachment of phosphatidylethanolamine to the ATG8 protein (Ichimura et al., 2004).

Specifically, the data generated herein were used to evaluate new insights concerning these two autophagy genes (i.e., *ATG7* and *ATG9*). But more broadly for understanding processes in plant system, the aim of the study was to assess how accurately multi-omics data (i.e., integrated transcriptomics and metabolomics data) can be used to predict gene functionalities without *a priori* knowledge concerning the specific functions of genes.

## Results

### Experimental platform to explore relationships between gene expression, lipids and autophagy

The experimental platform schematically illustrated in **Supplemental Figure 1**, was designed to compare three *Arabidopsis* genotypes, WT and two mutants that are blocked in autophagy (i.e., *atg7* and *atg9*), all in the Col-0 ecotype background. These genetic stocks were grown in normal conditions (i.e., (+N)-condition) and in conditions that were designed to induce autophagy, by depriving nitrogen from the growth medium (i.e., (-N)-condition). Three days after this environmental induction of autophagy, seedlings were harvested in triplicate for lipidomics and transcriptomics analyses. The harvested seedlings were separated into the root and aerial tissues, which were separately analyzed in order to evaluate if autophagy is distinct between these two organs. Initially a small segment of the roots was stained with monodansylcadaverine and examined by fluorescence microscopy to visualize autophagosomes (Contento et al., 2005), which confirmed that the autophagy process was induced in the WT plants by the (-N)-treatment, whereas this process was inhibited in the *atg7* and *atg9* mutants (Hanaoka et al., 2002; Thompson et al., 2005). Regardless of the seedling genotype, all seedlings showed an obvious yellowing in the autophagy-inducing condition, which began to appear approximately 48-hours after the (-N) treatment.

### Changes in patterns of gene expression

Sequencing of the RNA and statistical analysis of the read-counts from each of the resulting tissue samples identified the differentially expressed genes (DEGs) whose expression was affected by the genetic and/or environmental alteration in the autophagic state of the seedlings. The statistical significance of these comparisons was evaluated at two levels of significance, q<0.05 and q<0.1 (**Table 1**; **Supplemental Table 1**). The entire list of DEGs identified by these comparisons are provided in **Supplemental Table 2**. Because the evaluations using a statistical significance level of q<0.1 generated more comprehensive lists of DEG that ultimately provided higher confidence in the GO enrichment analysis, all subsequent evaluations of DEGs were conducted using this latter statistical significance level.

**Table 1:** Number of differentially expressed genes in leaves and roots of WT, and *atg7* and *atg9* mutants grown in normal (+N) or autophagy-inducing (-N) conditions.

	Leaf				Root			
	Up-regulated		Down-regulated		Up-regulated		Down-regulated	
Comparison	Genome-wide^a^	Lipid genes^b^	Genome-wide	Lipid genes	Genome-wide	Lipid genes	Genome-wide	Lipid genes
1 WT(-N)/WT(+N)	3768	122	2994	117	1850	112	1574	35
2 atg7(-N)/atg(+N)	4355	160	3974	116	3074	176	2157	38
3 atg9(-N)/atg9(+N)	1881	82	1332	45	2186	125	1508	28
4 atg7/WT, (+N)	18	1	6	0	95	2	152	11
5 atg7//WT, (-N)	2301	98	2741	49	812	48	397	13
6 atg9/WT, (+N)	11	0	23	1	19	1	38	2
7 atg9/WT, (-N)	86	4	264	6	38	0	45	4

a Among all annotated genes in the Arabidopsis genome that show statistically significant differential expression (At a statistical significance level of <0.1).

b Among the lipid metabolism associated genes identified in the AraLip database (http://aralip.plantbiology.msu.edu) that show statistically significant differential expression (At a statistical significance level of <0.1).

These comparisons indicate that the environmental condition that stimulated autophagy (i.e., (-N)-treatment) induced major changes in gene expression among all genotypes and organs. The *atg7* mutant showed the largest number of DEGs in the (-N)/(+N) comparison (~8,300 DEGs in leaves and ~5,200 DEGs in roots), as compared to the numbers observed in the WT genotype (~6,700 DEGs in leaves and ~3,400 DEGs in roots). In contrast, the *atg9* mutant showed the lowest number of DEGs in this comparison (~3,200 DEGs in leaves and ~3,700 in DEGs in roots). In all these comparisons, the number of up-regulated DEGs were 10-40% greater than the number of down-regulated DEGs.

The conclusions based on the (-N)/(+N) induced changes in the transcriptomes are reinforced when we compared the number of DEGs observed in the *atg7* vs. WT and *atg9* vs. WT comparisons in the (-N) condition. Namely, the *atg7* mutation has the larger effect in altering the transcriptome (i.e., ~5000 and ~1200 DEGs in leaves and roots, respectively), than the *atg9* mutation (i.e., 350 and<100 DEGs in leaves and roots, respectively). These rather large differences in the altered transcriptomes between the two mutants and WT, which are induced by the (-N) treatment, contrast to the situation we found when these comparisons were made in the (+N) condition. Specifically, in the (+N) condition there were only 24 and ~250 DEGs in leaves and roots, respectively, between *atg7* and WT plants, and for the *atg9* mutant these respective numbers were 34 and 58 DEGs. In most of these comparisons (with the exception of the *atg9* mutant), the leaf tissue is considerably more responsive than the root tissue to changes in the (-N)-treatment and/or genotype.

**Figure 1:**
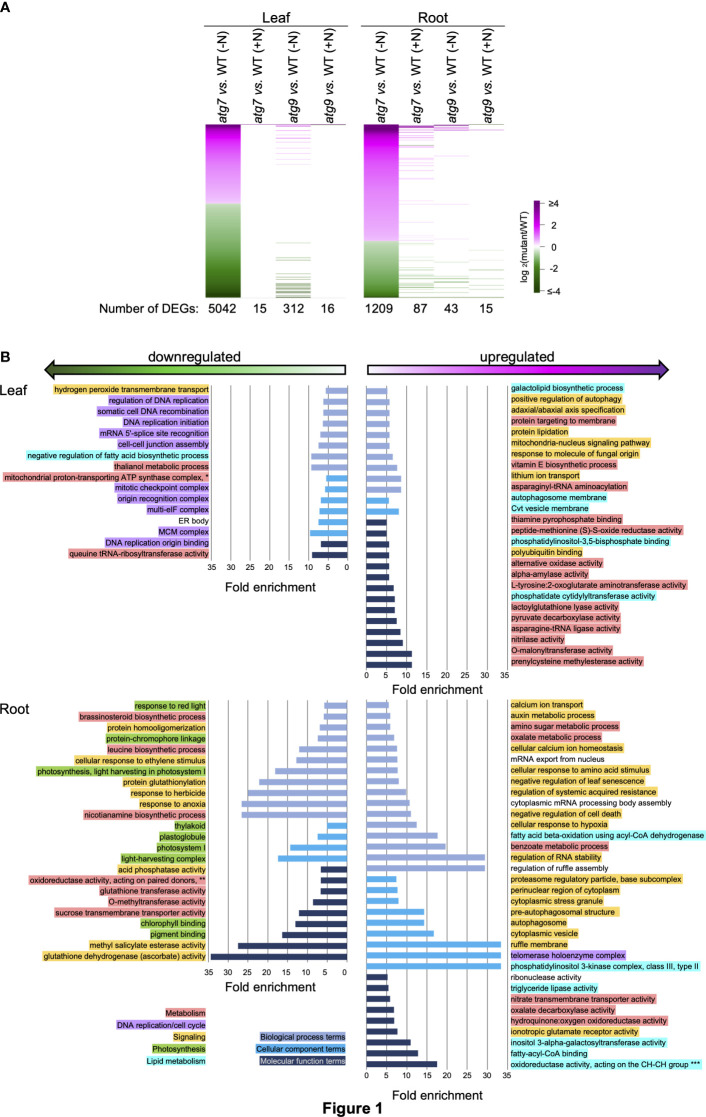
Biological functionalities identified by DEGs induced by the *atg7* mutation in leaves and roots. **(A)**. Heat map visualization of the relative expression of 5042 and 1209 DEGs identified by comparing transcript abundances in leaves and roots of the *atg7* mutant versus WT grown in the (-N) condition. The expression of each of these DEGs is compared to their relative expression levels in the other indicated comparisons. The color scale indicates the relative expression level of each DEG in each comparison. **(B)**. Using the list of upregulated and downregulated DEGs for leaves and roots, GO terms that show >5-fold enrichment were generated by the sequential use of DAVID and REVIGO. The GO terms that are unique to the altered transcriptome of the leaves and roots are presented. GO enrichment terms that are shared between the leaves and roots are presented in **Supplemental Figure 2**. The graphs plot the fold-enrichment for each GO enrichment terms associated with Biological Processes (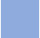), Cellular Component (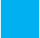) and Molecular Function (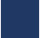). Each GO enrichment term is categorized with higher level functionalities associated with metabolism (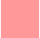), DNA replication (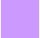), signaling (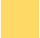), photosynthesis (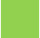) and lipid metabolism (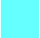). GO terms: *mitochondrial proton-transporting ATP synthase complex, coupling factor F(o) **oxidoreductase activity, acting on paired donors, with incorporation or reduction of molecular oxygen, 2-oxoglutarate as one donor, and incorporation of one atom each of oxygen into both donors ***oxidoreductase activity, acting on the CH-CH group of donors, with a flavin as acceptor.

**Figure 2:**
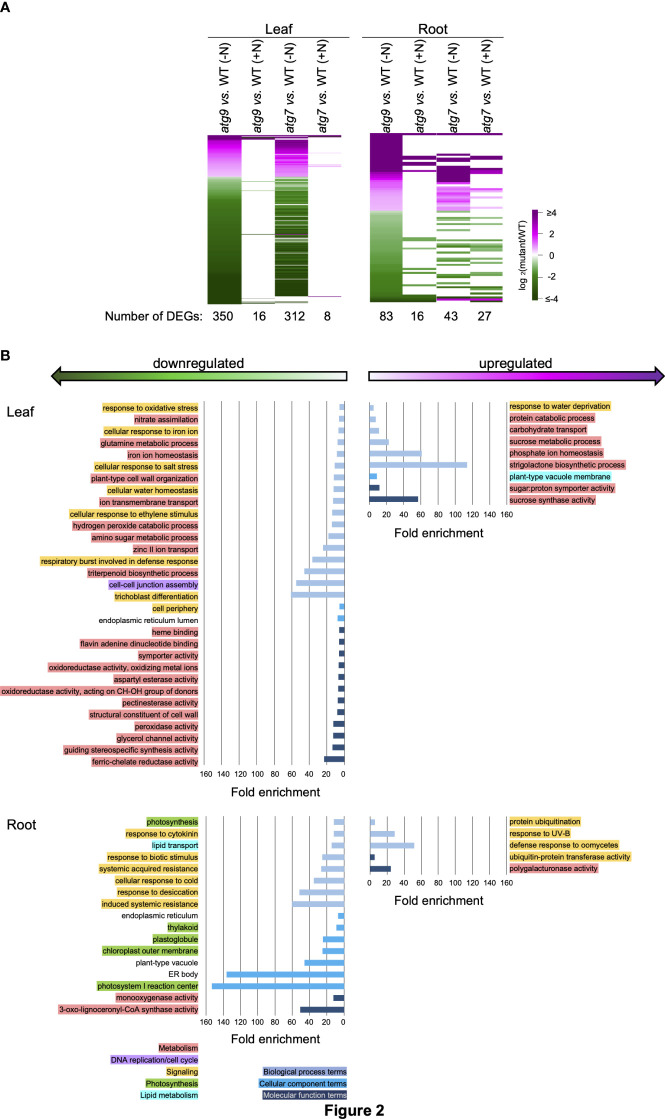
Biological functionalities identified by DEGs induced by the *atg9* mutation in leaves and roots. **(A)**. Heat map visualization of the relative expression of 350 and 83 DEGs identified by comparing transcript abundances in leaves and roots of the *atg9* mutant versus WT grown in (-N) condition. The expression of each of these DEGs is compared to their relative expression levels in the other indicated comparisons. The color scale indicates the relative expression level of each DEG in each comparison. **(B)**. Using the list of upregulated and downregulated DEGs for leaves and roots, GO terms that show >5-fold enrichment were generated by the sequential use of DAVID and REVIGO. The GO terms that are unique to the altered transcriptome of the leaves and roots are presented. GO enrichment terms that are shared between the leaves and roots are presented in **Supplemental Figure 2**. The graphs plot the fold-enrichment for each GO enrichment terms associated with Biological Processes (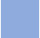), Cellular Component (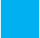) and Molecular Function (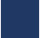). Each GO enrichment term is categorized with higher level functionalities associated with metabolism (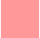), DNA replication (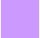), signaling (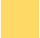), photosynthesis (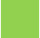) and lipid metabolism (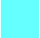).

There is very small overlap among the lists of DEGs that respond to the (-N)-treatment between the *atg7* mutant and the *atg9* mutant. Namely, of the ~5000 DEGs that are detected in the *atg7* (-N) vs WT (-N) leaf comparison, only 312 also occur in the *atg9* (-N) vs WT (-N) leaf comparison (**Figure 1A**). The same conclusion is drawn from the comparison of root transcriptomes; namely, of the ~1200 DEGs in the *atg7* (-N) vs WT (-N) comparison, only 43 also occurred in the *atg9* (-N) vs. WT (-N) root comparison (**Figure 1A**). However, when we considered the smaller number of DEGs that are detected in the *atg9* (-N) vs WT (-N) comparison, a large majority of these (between 50% and 90%) are also DEGs in the *atg7* (-N) vs WT (-N) comparisons of both roots and leaves (**Figure 2A**). It should be noted that a small number of these latter DEGs (<10) switched from being induced or suppressed in one mutant to being suppressed or induced, respectively, when one compares their expression in the other mutant.

Functional insights from these DEG lists were gained by conducting GO enrichment analysis *via* the sequential use of the annotation pipelines, DAVID (Huang et al., 2009; d. W. Huang and Lempicki, 2009) and REVIGO (Supek et al., 2011). These analyses identified 177 GO enrichment terms that are >5-fold enriched among the down-regulated and upregulated DEG lists. These terms were further categorized as being either common between the two tissues (i.e., roots and leaves) or they are uniquely associated with either the root or leaf tissue. **Supplemental Figure 2** identifies the 15 GO terms that are common to both leaves and roots, and these include “secondary metabolic process” and “autophagy” terms in *atg7* vs. WT comparison, and the “lipid binding” term in *atg9* vs. WT comparison. **Figures 1B**, **2B** show the GO terms that differed among the two tissues (i.e., leaves and roots). These GO terms were further categorized into five higher level functional annotations, i.e., metabolism, DNA replication/cell cycle, signaling, photosynthesis or lipid metabolism, and these higher order functionalities are color coded in **Figures 1B**, **2B**. These analyses indicate that the *atg7* and *atg9* mutations affect different down-stream processes, and in each mutant the processes that are altered in leaves are different from those that are altered in roots. Specifically, in the *atg7* mutant the upregulated DEGs in leaves are enriched with GO terms associated with metabolism and signaling functionalities, whereas the down-regulated DEGs in this organ are associated with DNA replication/cell cycle functionalities (**Figure 1B**). The GO terms that are categorized in the signaling functionality identify processes that are specifically associated with autophagy (e.g., pre-autophagosomal structure and autophagosome) and with a combination of stresses that are possibly mediated by changes in the oxidative status of the root tissue (e.g., hypoxia and anoxia, glutathione and ascorbate metabolism, and responses associated with cell death, senescence, stress granules, and herbicides). Similar analyses of the GO terms identified by the DEGs in the *atg9* mutant indicate that metabolism functionalities are affected in the leaves, whereas in the roots the predominant functionality that is affected is signaling (**Figure 2B**).

### Changes in the transcriptome in response to autophagy

By combining the comparisons of the transcriptomes of the *atg7* and *atg9* mutants to those of WT plants that were grown in either the normal, (+N)-condition or the autophagy-inducing, (-N)-condition, we were able to distinguish transcriptomic changes that occurred because of the change in the N-status of the plants from those that are associated with the induction of autophagy. This identification depended on the fact that the *atg7* or *atg9* mutations block autophagy, and therefore we reasoned that the changes in the transcriptome that occurred in response to the (-N) or (+N) treatment of either the *atg7* or *atg9* mutants that were different from those observed for the WT plants are associated with the process of autophagy. This strategy is schematically illustrated in the expression ratio plots shown in **Supplemental Figure 3A**. Specifically, the plot illustrates the expression ratio of a hypothetical gene in the (-N)/(+N) comparison in WT plants (green datapoints), and in autophagy mutant plants (orange datapoints). Genes that show an expression ratio where the green and orange datapoints are indistinguishable from each other, but show altered expression due to the (-N) treatment, were identified as responding to the (-N)-treatment of the plants but not altered by autophagy (**Supplemental Table 4C**). All other combinations of gene expression ratios, where the green and orange datapoints are statistically distinguishable from each other (q<0.1), are changing their expression levels in response to the autophagic state of the plants. These autophagy-responding genes can be further classified into three categories: a) those genes that showed enhanced differential expression in the autophagy mutant compared to the WT; b) those genes that showed reduced differential expression in the autophagy mutant compared to the WT; and c) those genes that showed opposite differential expression in the autophagy mutant than in the WT.

**Figure 3:**
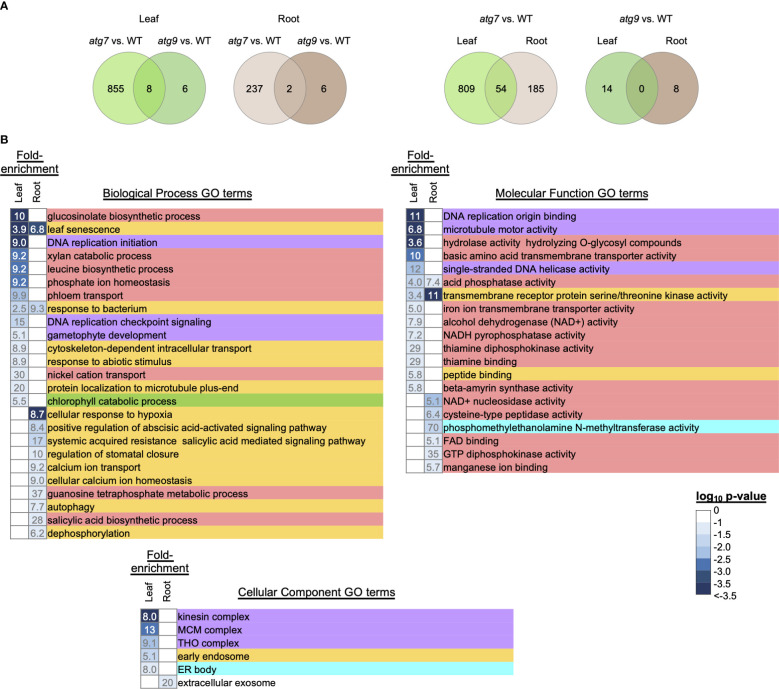
GO enrichment analysis of genes that are differentially expressed upon alterations in autophagy. DEGs that are responding to the alteration in autophagy were identified by the strategy described in **Supplemental Figure 3**. Venn diagrams identify the overlap of the autophagy responsive DEGs that were due to the *atg7* or *atg9* mutations in either leaves or roots **(A)**. The leaf and root GO terms showing >5-fold enrichment were identified by using the list of autophagy responsive DEGs that were responding to the *atg7* mutation **(B)**. The intensity of the blue color shading of the fold-enrichment data is proportional to the log_10_ p-value for each GO term as determined by DAVID. Each GO enrichment term is color coded to indicate higher level functional category associated with metabolism (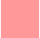), DNA replication (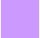), signaling (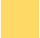), photosynthesis (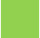) and lipid metabolism (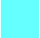).

Using this integrated comparison strategy, we identified 863 DEGs in leaves and 239 DEGs in roots whose expression is responding to the *atg7*-defect in autophagy. In parallel we identified 14 DEGs in leaves and 8 DEGs in roots whose expression is responding to the *atg9*-defect in autophagy (**Figure 3**; **Supplemental Figure 3B**). Thus, by integrating these comparisons we were able to distinguish the smaller subset of autophagy-responding DEGs from the larger number of DEGs that are responding to the change in the N-status of the plants (**Table 1**). The functionalities of the genes that are responding to the (-N)-treatment (**Supplemental Figures 4**–**6**) or to the autophagy defects (**Figure 3**) were explored by conducting GO enrichment analysis.

Consistent with prior studies, which indicate that the *atg7* mutant has a more severe phenotypic defect than the *atg9* mutant (Shin et al., 2014), we found a larger number of DEGs that are responding to the *atg7*-defect in autophagy than the number responding to the *atg9*-defect in autophagy. The Venn diagrams shown in **Supplemental Figure 3B** indicate that there is very little overlap among the autophagy-responsive genes in the *atg7* and *atg9* mutations, and similarly, there is a very small overlap in these autophagy-responsive genes between leaf and root tissues. These findings suggest that in the absence of either *ATG7* or *ATG9* gene-functions there are distinct down-stream effects, which may also be reflective of the differential expression pattern of these genes between leaves and roots.

Because of the small number of genes whose expression is affected by autophagy due to the *atg9* mutation (22 DEGs), it was difficult to further assess functionalities that are associated with this genetic defect. However, such analyses were pursued with the gene list identified from the *atg7* mutant (**Figure 3A**). Only a small fraction (~5%) of these autophagy-responsive DEGs occur in both leaves and roots, and the majority (~80%) are unique to leaves. Using the list of leaf and root DEGs as inputs we conducted GO term enrichment analysis to identify a broad overview of the types of processes that are affected by autophagy. These analyses identified 92 GO terms that show statistically significant enrichment (p< 0.1) (**Supplemental Figure 7**), and **Figure 3** focuses on those GO terms that show more than 5-fold enrichment. Comparison of the GO terms recovered with the list of leaf and root DEGs indicates that different processes are being affected by autophagy in the two organs. This difference is most pronounced with the GO Biological Process terms, which are populated by signaling processes in roots, whereas in leaves they are populated by metabolism associated processes. This enrichment of metabolism associated terms is also apparent in the GO Molecular Function category.

### Changes in the lipidome

**Figure 4:**
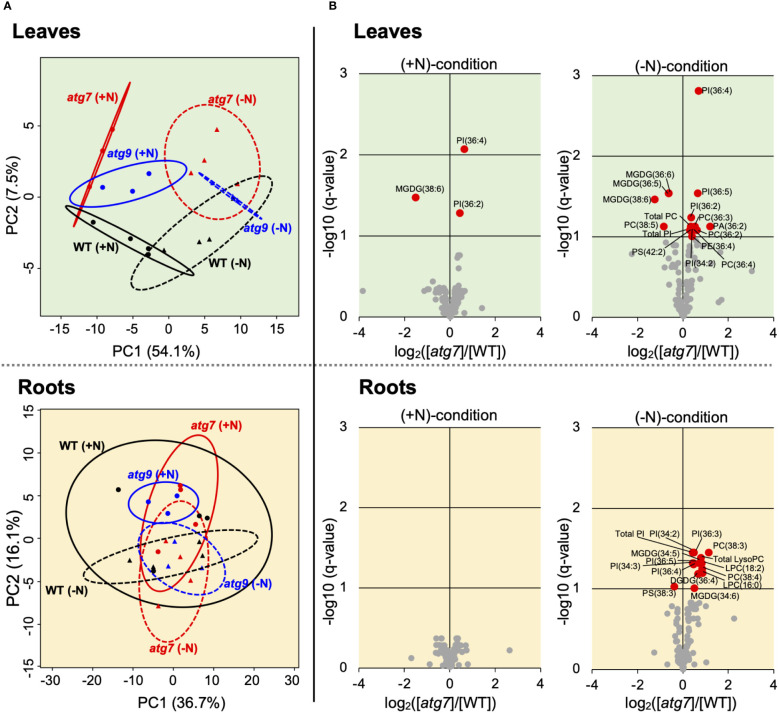
Changes in the leaf and root lipidomes induced by the *atg7* or *atg9* mutations and modified by (-N) treatment. PCA analysis of lipidomics data obtained from leaves and roots of WT (n=4), *atg7* (n=4) and *atg9* (n=3) mutant plants grown in either (+N) or (-N) conditions. The 95% confidence ellipses of each treatment are indicated **(A)**. Volcano plot visualization of the differences in the leaf and root lipidomes between WT and *atg7*-mutant grown in (+N) or (-N) conditions. q-values were obtained from p-values, which were based on the Wald test (n = 4). Red-colored datapoints represent statistically significant difference in accumulation of specific lipids **(B)**. Parallel volcano plots of the *atg9* mutant data are presented in **Supplemental Figure 8**.

Lipidomics data were gathered in parallel to the described transcriptomics data. These analyses identified 156 specific lipid species, which included LysoPC, LysoPE, LysoPG, PC, PA, PG, PE, PI, PS, MGDG and DGDG. Upon statistical analyses, we obtained rigorous data on the abundance changes for 97 lipid molecules. PCA analyses of the leaf lipidomics data indicates that the major driver that separates the lipidomes of these samples is associated with the nitrogen treatment of the seedlings (**Figure 4A**, **Supplemental Figure 8**). Namely, PC1 that accounts for ~54% of the variance in the data, separates samples based upon whether they were grown on (+N) or (-N) media. Separation of the sample by genotype is captured by PC2, and accounts for only 7% of the variance in the data.

**Figure 4B** illustrate that there are only a limited number of lipids species whose abundances are significantly affected by the *atg7* mutation (3 and 14 specific lipids in the (+N) and (-N) conditions, respectively), and these appear to be enriched with lyso-PC, PC, PI and MGDG lipids. As with leaf tissue, the effect of the *atg7* mutation on the root lipidome was more evident in the (-N) treatment, with the abundances of 13 specific lipids being altered in the mutant (**Figure 4B**), whereas in the (+N) condition, no lipid species where affected by the *atg7* mutation (**Figure 4B**). Comparison of the lipidomes of roots and leaves indicate that the *atg7* mutation induced different changes in the two organs; only ~15% of the altered lipids are shared between leaves and roots. In contrast to the effect of the *atg7* mutation on the leaf and root lipidomes, the *atg9* mutation did not affect a detectable change in the lipidomes of these organs, irrespective of whether the plants were grown in the nitrogen replete or nitrogen deficient condition (**Supplemental Figure 8**).

### Spatial distribution of specific lipids

**Figure 5:**
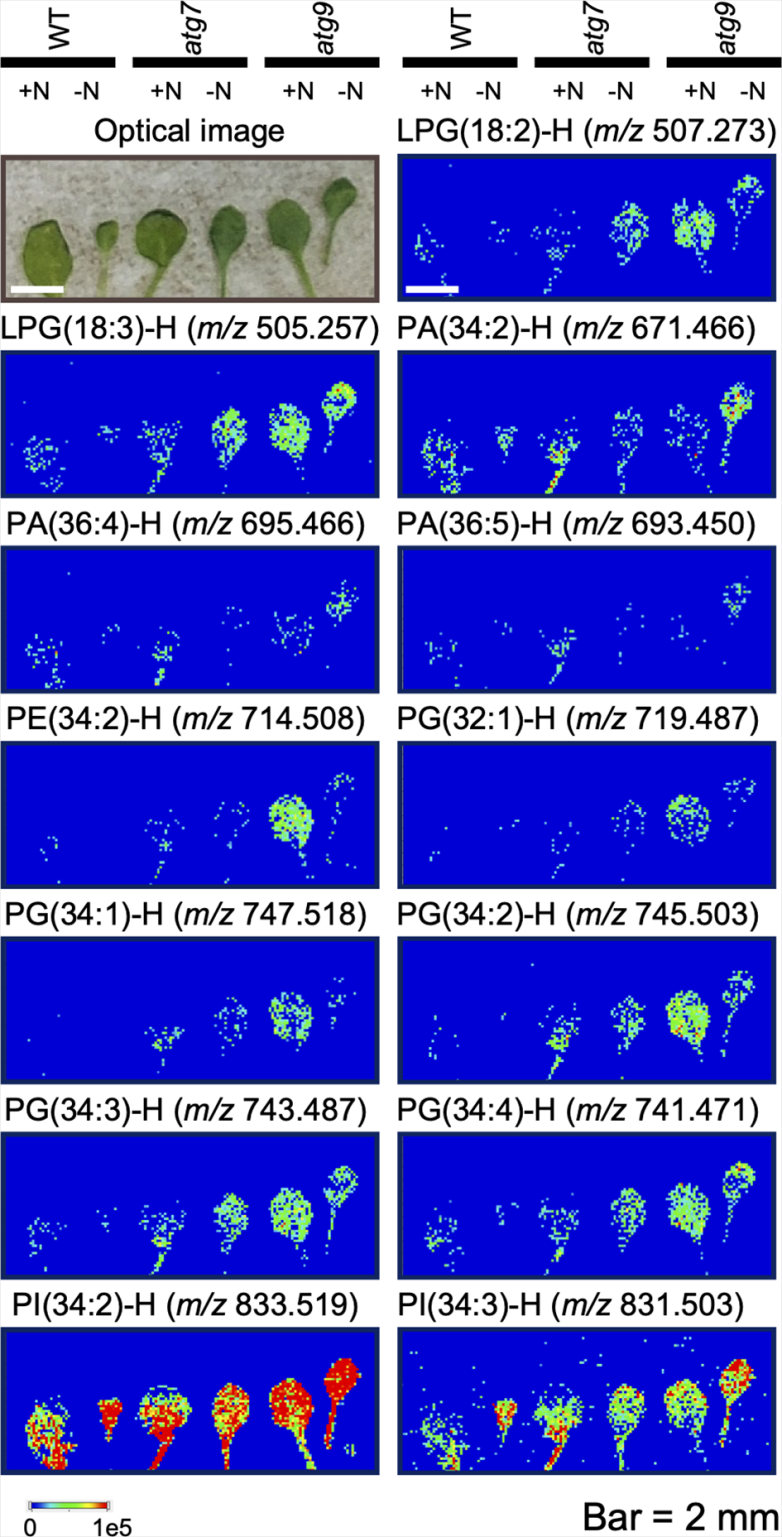
Mass spectrometric imaging of the spatial distribution of specific phospholipids in WT, *atg7* and *atg9* mutant leaves. One of the first pair of true leaves from plants grown in either (+N) or (-N) treatment of the indicated genotypes were used in these imaging experiments. Leaves were longitudinally fractured to reveal the leaf interior, and this exposed surface was imaged optically or by mass-spectrometry. All mass-spectrometric images detected the deprotonated ion ([M−H]^−^) of the indicated phospholipid molecular species in negative mode using 1,5-diaminonaphthalene (DAN) as the matrix. The colored scale bar shows the relative intensities within a sample. The ion signals were normalized to the total ion count and the maximum values of 1x10^5^ was used for all the images.

We used MALDI-MSI to query the spatial distribution of specific lipids in the organs from WT and the autophagy mutants. Initial studies evaluated both roots and leaves of these plants grown either in the (+N) or (-N) conditions. Unfortunately, examination of root cross-sections by MSI proved technically difficult, probably due to the low abundance of lipids in this organ, and only a limited set of data could be collected (**Supplemental Figure 10**). Therefore, a complete set of MS images were collected from the leaves of WT, and *atg7* and *atg9* mutant plants that were grown either in the (+N) or (-N) conditions. To ensure reproducibility of the data, all images were generated from at least three different biological samples, and **Supplemental Figure 9** shows images of an exemplary set of triplicate data of the lipid, LysoPG(18:2). Similar reproducibility was obtained with all the lipid images, and **Figure 5** shows representative images for 13 distinct phospholipids. These lipid distribution images were selected to ensure we determined the spatial distribution of a wide range of phospholipids, and warranted by a robust imaging signal, which ensured reliable reproducibility in the collected data. These data indicate that of the lipids that were imaged, there is no apparent differential distribution of each phospholipid molecule among the cell populations of these leaves, irrespective of the autophagic status of the seedling determined by the combination of genotype and nitrogen treatment.

### Changes in the expression of lipid metabolism associated genes

**Figure 6:**
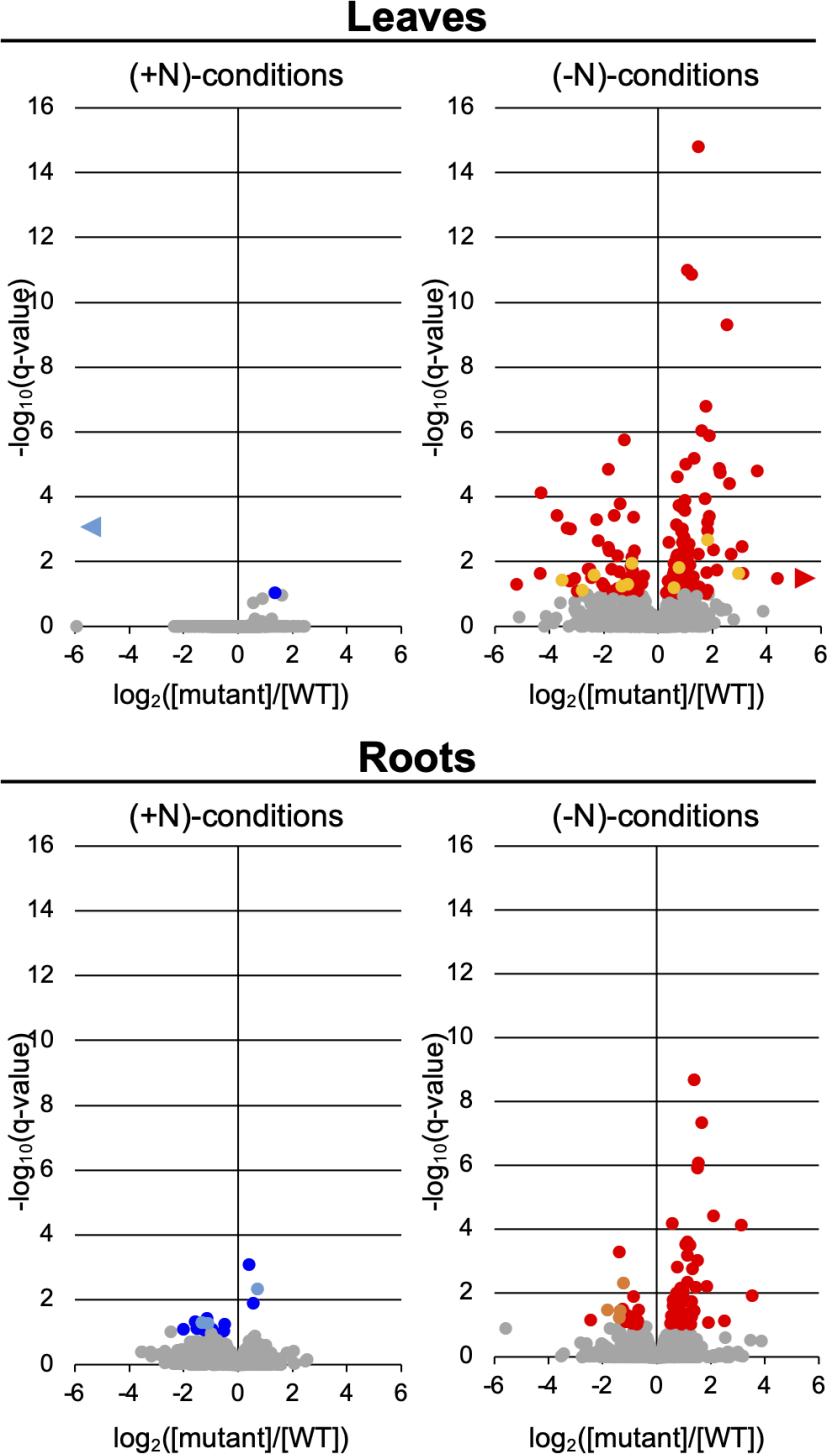
Changes in expression of lipid metabolism genes. Volcano plot visualization of lipid metabolism DEGs, comparing relative abundance of individual transcripts in the *atg7*- (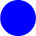, 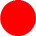) or *atg9*-mutant (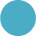, 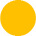), in leaves and roots from plants grown either in (+N)-or (-N)-conditions. Grey data-points (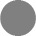) represent transcripts whose relative abundance is statistically indistinguishable in these comparisons (q > 0.1). Data points that are out of the range of the x-axis are identified by horizontal isosceles triangles (►), which point to the direction of the data-point, either to the left or right of the zero x-axis value. Lipid metabolism genes were identified in AraLip (Li-Beisson et al., 2013).

Gene loci encoding functions associated with lipid metabolism in *Arabidopsis* are categorized in the AraLip database (http://aralip.plantbiology.msu.edu/) (Li-Beisson et al., 2013). AraLip identifies 775 *Arabidopsis* genes that have either been demonstrated to be involved in lipid metabolism or are hypothesized to be associated with such processes. We examined the transcriptome data to specifically identify those lipid genes whose expression were affected by either the *atg7* or *atg9* mutation in the context of the nitrogen treatment. These analyses identified 197 lipid genes (i.e., the altered lipid transcriptome) whose expression was significantly affected by either the *atg* mutations or the nitrogen treatment (**Figure 6**; **Supplemental Table 3**). The altered lipid-related transcriptome is larger in the *atg7* mutant than in the *atg9* mutant, and in both mutants the size of this altered transcriptome is increased significantly by the (-N) treatment (**Figure 6**); specifically, there are 184 lipid-related DEGs in the *atg7* mutant, and only 13 such DEGs in the *atg9* mutant. This parallels the changes we observed in the lipidome of the *atg7* and *atg9* mutant seedlings, with hardly any detectable changes in the lipids of the latter mutant (**Figure 4**; **Supplemental Figure 8**). Moreover, the size of the altered lipid-related transcriptome is larger in the leaves than in the roots [i.e., more DEGs in leaves (146 genes) than roots (75 genes)] (**Figure 6**). In the case of the *atg7* mutant grown in the (-N) condition, where the altered lipid-related transcriptome is sufficiently large to provide statistical rigor, the number of the DEGs that are upregulated is between 2- and 4-fold larger than the number of down regulated genes, in both leaves and roots.

Insights into the lipid-related gene functionalities that were affected by these treatments were gleaned by identifying the lipid pathways pinpointed by the DEGs (**Supplemental Table 3**). These annotations identified 15 lipid metabolic pathways, which can be classified as either a) biosynthetic (i.e., fatty acid biosynthesis, elongation and metabolism, chloroplastic lipid metabolism, cuticle and wax esters deposition and triacylglycerol biosynthesis); b) catabolic (i.e., fatty acid degradation, triacylglycerol metabolism and lipases), c) membrane lipid reorganization (i.e., lipid transport and binding proteins, and phospholipid metabolism) and d) signaling lipids (i.e., phosphoinositide, oxylipin and sphingolipid metabolism). Because the affected lipid-related transcriptome is larger in the *atg7* mutant than the *atg9* mutant, we were able to surmise that in the leaves and roots of the former mutant, the down-regulated genes in the (-N) treatment are enriched with lipid biosynthetic pathways and pathways that reorganize membrane lipids In contrast, the up-regulated genes are enriched in processes associated with lipid catabolism and the metabolism of signaling lipids.

## Discussion

The premise for this study was to assess the ability of multi-omics data, specifically integrated lipidomics and transcriptomics data, to enable the deduction of gene function without specific *a priori* knowledge concerning the biochemical function of the gene product(s). We focused the study on two genes, *ATG7* (At5g45900) and *ATG9* (AT2G31260), which are known to be components of the autophagic system of plants because they are homologs of the yeast genes involved in this process, and mutations in these two *Arabidopsis* genes block the process of autophagy. In yeast approximately 40 *ATG* genes have been characterized, and the molecular model of the process of autophagy is primarily based on the characterization of these genes and gene products (Farre and Subramani, 2016; Majeed et al., 2022). Multi-omics studies of *Arabidopsis atg* mutants have been interpreted in the context of this autophagy model, and this includes for example the characterizations of *atg4, atg5*, *atg9, atg7 and atg18* mutants (Masclaux-Daubresse et al., 2014; Avin-Wittenberg et al., 2015; Havé et al., 2019).

The *Arabidopsis atg7* mutants are completely blocked in autophagy and are hypersensitive to shortage in nutrients (Doelling et al., 2002), and carbon- and N-deficiency (Thompson et al., 2005; Chung et al., 2010). Prior detailed biochemical characterizations of *ATG7* have established that it encodes an enzyme that is similar to the ATP-dependent, E1 ubiquitin-activating enzyme, which catalyzes the covalent conjugation of ATG5 and ATG12, a process required for autophagy (Mizushima et al., 1998). The resulting ATG5-ATG12 conjugate connects to the dimeric ATG16 protein, and the complex is incorporated into the phagophore, allowing for lipidation of ATG8 with a phosphatidylethanolamine molecule (Ichimura et al., 2000; Hanada et al., 2007; Chung et al., 2010). These biochemical processes result in the recruitment of ATG8 and cargo macromolecules into autophagosomes, and sealing of the new organelle (Li and Vierstra, 2012; Bao et al., 2017; Martens and Fracchiolla, 2020).

The *ATG9* gene encodes the only integral membrane protein of the core autophagy machinery, and it functions in the development and expansion of the autophagosome (Lang et al., 2000). Mutations in the *ATG9* gene results in the failure to release autophagosomes from the ER (Zhuang et al., 2017), which is probably due to the inability to incorporate membrane lipids into the developing organelle. This supposition is based on *in vitro* characterization of ATG9 that identified it as a scramblase (Maeda et al., 2020; Orii et al., 2021), which together with ATG2 (Maeda et al., 2019) incorporates preexisting ER-derived phospholipids and newly synthesized lipids into the nascent and growing autophagosome (Schütter et al., 2020). This mechanistic understanding of ATG9 function is based on structural characterization of ATG9-orthologs from *Arabidopsis* (Lai et al., 2020), yeast (Matoba et al., 2020) and humans (Guardia et al., 2020), which is indicative of the phylogenetic conservation of the autophagic process across a wide range of species (Zhang et al., 2021).

The molecular characterization of mutants by a multi-omics systems biology strategy, as conducted in this study, cannot provide analogous mechanistic insights on gene-function. Rather, the lipidomics and transcriptomics data that have been presented enable more detailed molecular depiction of the effect of each mutation, namely the molecular phenotype of the mutations. Moreover, the biochemical mechanistic understanding of the function of the *ATG7* and *ATG9* genes products provides the basis for proposing a more comprehensive physiological model to explain the consequence of the disruption in each of these gene functions. Hence, understanding the physiological function of genes requires the need for detailed biochemical characterizations of gene products, and the multi-omics data associated with the characterization of strains that are missing those genetic functions. Additional physiological insights could also be gained when these gene products are overexpressed, although such genetic manipulations do not always impact changes in the molecular phenotype. Previous omics-type studies support the idea that it is difficult to generalize about function from genome-scale data due to indirect effects that may differ among different genotypes and species. For example, in maize, a mutant defective in autophagy presents more molecular phenotypes in nitrogen-replete conditions than in nitrogen-deficient conditions (McLoughlin et al., 2018), whereas our analysis and a previous study using adult *Arabidopsis* mutant plants (Masclaux-Daubresse et al., 2014), found more molecular phenotypes upon nitrogen deficiency. Therefore, although there appear to be different responses in lipid profiles associated with the genetic and environmental disruption of autophagy, these differences require a mechanism of lipid recycling, which has previously been reported in both maize and *Arabidopsis* autophagy mutants (McLoughlin et al., 2018; Havé et al., 2019).

Specifically, the lipidomics and transcriptomics data generated within this study provide insights on the molecular response of *Arabidopsis*, downstream of the genetic block associated with the *atg7* and *atg9* mutations. The initial observation from these studies was the finding that the *atg7* mutation has a greater impact than the *atg9* mutation on the assessed transcriptome and lipidome. Moreover, this difference in the magnitude of the molecular response to each of these mutations was reflected in both organs that were assessed, i.e., leaves and roots. Indeed, the leaves showed a larger molecular response to these mutants than the roots. Although, the magnitude difference in the morphological phenotypes of the *atg7* and *atg9* mutants had previously been noted (Shin et al., 2014; Kang et al., 2018), the mechanism to explain this difference is not clear. This is particularly intriguing considering that the *atg9* mutation is thought to directly affect lipid-related processes, as it is required for incorporation of lipids into autophagosomes and therefore for autophagosome expansion. Despite this direct connection, the *atg9* mutant showed very little change in lipid profile, and also in lipid-related transcriptome, when compared with the *atg7* mutant. As a linear pathway cascade, it might be expected that a genetic block anywhere in the autophagy pathway should generate similar downstream responses. Yet, not only is the magnitude of the response different between the two mutants, but as visualized by GO enrichment analysis, the *atg7* and *atg9* mutations affected different processes, and these differences are also sustained as related to different organs (i.e., leaves and roots), but not the different tissues of the leaf. Specifically, as revealed by the MSI experiments, the cellular distribution of the lipids that were imaged in the leaves of *Arabidopsis* is not differentially affected by either of the two mutants that were studied.

There are a number of potential explanations for the difference in the downstream responses between the *atg7* and *atg9* mutants. A relatively trivial explanation may be associated with the nature of the mutant alleles that were used in these experiments. For example, the weaker phenotype of the *atg9* mutant maybe due to the fact that the *atg9-4* (SALK_145980) allele (Floyd et al., 2015) used in this study is not a null allele and thus any remaining *ATG9* activity in the mutant is sufficient for the expression of a weak phenotype. Molecular characterizations have identified *atg9-4* as a sequence-indexed T-DNA insertion allele (signal.salk.edu/cgi-bin/tdnaexpress) (Alonso et al., 2003), with the insertion located in the 7^th^ exon of the *ATG9* locus. Although it is possible that this allele can express a truncated ATG9 protein that would have some residual activity, we postulate that this is unlikely because characterization of the *atg9-3* allele (SALK_128991), which is disrupted by a T-DNA insertion at an upstream exon position, also shows a similar “weak” phenotype (see phenotype data concerning the *atg9-3* allele deposited at TAIR (Reiser et al., 2017). Another possibility is that the *ATG9* functionality may be redundant in the pathway, possibly as a consequence of another *ATG9*-like paralog in the *Arabidopsis* genome; however BLAST analysis of the genome indicates that such a paralog is not identifiable. There is a possibility however that the *ATG9-*function maybe provided by protein(s) that does not share sequence homology with the ATG9 protein. For example, flippases and floppases catalyze reactions, which collectively are equivalent to the scramblase reaction that ATG9 is thought to catalyze (i.e., lipid scramblase). An alternative explanation maybe associated with the observations that different ATG proteins, and therefore different branches of the autophagy pathway, maybe responding to different environmental signals that induce autophagy (Huang et al., 2019; Aroca et al., 2021). Indeed this possibility may also provide the explanation to account for the differences in the molecular phenotypes that are apparent between roots and leaves of the *atg7* and *atg9* mutants. We explored this possibility by evaluating both our own RNA-Seq data and the publicly available *Arabidopsis* transcriptome data (Sullivan et al., 2019). In the absence of any mutations, the *ATG9* transcript accumulates at ~50% higher levels than the *ATG7* transcript, irrespective of the organ. Consistent with their role in autophagy, the abundance of both the *atg7* and *atg9* transcripts is induced by ~2-fold in leaves, but such an induction does not occur in roots by treatments that induce autophagy. This lack of induction in the expression of these genes in roots, when autophagy is induced, may therefore be the basis for the relatively weak phenotype that is observed in this organ. Therefore, the reason that the autophagy response in the *atg9* mutant is weaker than the *atg7* mutant requires additional more mechanistic studies than those conducted herein.

Collectively, the observations reported in this study indicate that downstream of each of these two autophagy genes, the process of autophagy is differently expressed in different organs, and these differences manifest organ-specific phenotypes. Moreover, the multi-omics research strategy applied herein describes these phenotypes at the molecular level, rather than the more traditional whole organismal level. Yet, the explanation of the mechanism(s) that generate these molecular phenotypes requires *a priori* knowledge that can only be generated by the specific biochemical characterization of the gene products.

## Materials and methods

### Biological materials and growth conditions

Seed stocks of wild-type *Arabidopsis* (ecotype Col-0) and *atg7-2* (Chung et al., 2010) and *atg9-4* (SALK_145980) (Floyd et al., 2015) mutant lines were obtained from the Arabidopsis Biological Resource Center (Columbus, OH). Seeds were sterilized with 70% ethanol, followed by a 10-min incubation in 0.1% (v/v) Tween 20 (Thermo Fisher Scientific, Waltham, MA) and 50% (v/v) bleach solution. The seeds were then washed with sterile water, at least three times. Subsequently, the suspended seeds were vernalized by incubating at 4°C in darkness for 2 days.

After vernalization, the seeds were suspended in sterile 0.1% (w/v) agarose (VWR, Radnor, PA), and sown on 3.5 cm x 4 cm autoclaved stainless steel growth mesh (14 Mesh T304 Woven Stainless 0.017” wire diameter, TWP Inc. Berkeley, California), which was laid on ½ strength Murashige and Skoog (MS) solid medium composed of 2.15 g/L Murashige and Skoog Basal Salt Mixture (MilliporeSigma, Burlington, MA), 0.05% (v/v) Murashige and Skoog Vitamin Solution (MilliporeSigma), 1% (w/v) sucrose (Thermo Fisher Scientific), 6g/L Phytoblend Agar (Caisson Labs, Smithfield, UT), and 2mM MES (MilliporeSigma) at pH 5.7. Each 10 cm x 10 cm square Petri dish, containing 4 growth meshes, was placed in a growth room maintained at 22 °C under continuous illumination (50 ± 10 μE m^−2^ s^−1^) for a period of 5 days.

Subsequently, the growth mesh, carrying the germinated seedlings, was sterilely moved onto a sterile 7.5 cm x 8.5 cm stainless steel platform mesh (10 Mesh Woven Stainless 0.025” wire diameter, TWP Inc.), which was in a 11.4 cm × 8.6 cm × 6.4 cm Phytatray dish (MilliporeSigma) that contained sterile liquid medium, composed of ½ strength MS liquid media, which contained 10 mM NH_4_NO_3_ and 9.4 mM KNO_3_ (i.e., the (+N) condition). The volume of the medium was adjusted so that the growth mesh that carried the seeds was in contact with the surface of the medium, and thus as seedlings grew the root system extended into the liquid medium. After 1 day incubation in the +N liquid medium, half the growth meshes from each Phytatray dish were moved into a Phytatray dish that contained nitrogen-deficient liquid medium (-N medium, which contains no nitrogen salts). This medium was composed of 5% (v/v) Murashige and Skoog Basal Salt Micronutrient Solution (MilliporeSigma), 0.05% (v/v) Murashige and Skoog Vitamin Solution, 1.5 mM CaCl_2_, 0.75 mM MgSO_4_, 0.625 mM KH_2_PO_4_, 2.5 mM KCl, 2 mM MES and 1% (w/v) sucrose. After an additional 3-day incubation, the seedlings from both the +N and -N media were harvested by cutting the hypocotyls that were extending below the growth mesh, and leaf and root tissues were collected separately.

### Autophagy status verification by fluorescence microscopy

For MDC staining, *Arabidopsis* seedlings were stained with 50 mM monodansylcadaverine (MDC) (MilliporeSigma) in phosphate-buffered saline, pH 7.4 for 10 minutes, followed by 3 brief washes with phosphate-buffered saline, as previously described by (Contento et al., 2005). MDC-stained seedlings roots were imaged in the late elongation zone and neighboring cells in the differentiation zone, while root tips and older differentiation region cells were excluded. MDC fluorescence was visualized using a Zeiss Axio Imager.A2 upright microscope (Jean, Germany) using a 40X objective and a UV lamp and 4’,6-diamidino- 2-phenylindole-specific filter (DAPI). The number of motile MDC-stained puncta in all cells visible in the focal plane was quantified and expressed per frame for at least 10 images per sample for 3 biological replicates.

### RNA extraction

Excess moisture was removed from the collected fresh tissue by blotting the tissue with dry paper towels, then the tissue was transferred to 15 mL centrifuge tubes (Thermo Fisher Scientific), flash frozen with liquid N_2_, and stored at -80°C. About 1 g of harvested plant tissue was pulverized under liquid N_2_ in an RNase-free mortar and pestle. RNA was extracted with TRIzol Reagents (Thermo Fisher Scientific), followed by treatment with DNase I (QIAGEN, Hilden, Germany) and clean-up with RNeasy Mini Kit (QIAGEN). The quality of RNA was analyzed with an Agilent 2100 Bioanalyzer (Agilent Technologies, Santa Clara, CA). The RNA integrity number (RIN) of all samples was ≥7.8.

### RNA-Seq analysis

cDNA library construction and sequencing were conducted using a BGISEQ-500 system with 100-cycle paired-ends at BGI Americas (https://www.bgi.com/us/). Data files have been deposited in the NCBI Sequence Read Archive (https://www.ncbi.nlm.nih.gov/sra) under accession number PRJNA769614. The annotation file in the gff3 format and the genome DNA sequence of TAIR10 Release-39 were downloaded from http://ftp.ensemblgenomes.org/pub/plants/release-39/gff3/arabidopsis_thaliana/ and http://ftp.ensemblgenomes.org/pub/plants/release-39/fasta/arabidopsis_thaliana/dna/respectively. The splicing sites are extracted from the annotation file. The reads are aligned using HISAT2 (hierarchical indexing for spliced alignment of transcripts) (V2.1.0) (Kim et al., 2015) with known splice sites, mapped reads are counted by HTSeq-count (V0.9.1) (Anders et al., 2015) for raw counts and by cufflinks (V2.2.1) (Trapnell et al., 2010), for normalized counts.

### Lipid extraction

Lipids were extracted using a modification of a standard protocol (Shiva et al., 2018). The collected plant tissues (leaf or root) were transferred to a 50 mL Teflon-lined screw-capped glass tube (Thermo Fisher Scientific) containing 3 mL preheated isopropanol (Thermo Fisher Scientific) containing 0.01% (v/v) butylated hydroxytoluene (BHT) (MilliporeSigma) and 1 µM 1,2-didecanoyl-sn-glycero-3-phosphocholine (MilliporeSigma) as an internal standard. The tubes were incubated at 75°C for 15 min to quench the action of any lipases.Following the addition of 1.5 mL chloroform and 0.6 mL water the mixture was vigorously shaken at room temperature for 1 h. The clear liquid extract was transferred to another 50 mL tube using glass Pasteur pipettes, and the remnant tissue was further extracted for 30-minutes with another 4 mL chloroform/methanol (2:1) that contained 0.01% BHT. The clear liquid from this second extraction was removed and combined with the initial liquid extract. This chloroform/methanol (2:1) extraction was repeated three times, the last extraction being incubated overnight. The residue tissue remaining after lipid extraction was dried at 105°C, and the dry weight of each sample determined; each leaf tissue sample weighed approximately 20 mg, and each root tissue sampled weighed approximately 10 mg.

All extract aliquots from each biological sample were combined into a single screw capped tube and stored at -80°C under a nitrogen gas atmosphere. The solvent from each extract was removed by evaporation with the aid of a stream of N_2_ gas, and the lipid residue was dissolved in 1 mL chloroform and transferred to 2.0 mL clear glass vial with Teflon-lined screw cap (Thermo Fisher Scientific). The solvent was again evaporated with N_2_ gas, and the vials were shipped overnight on dry ice to the Kansas Lipidomics Research Center (https://www.k-state.edu/lipid/) for lipidomics analysis.

### Lipidomics analysis by ESI triple-quadrupole MS

Mass spectrometric analysis of the lipidome was conducted at the Kansas Lipidomics Research Center using a Xevo TQ-S mass spectrometer (Waters Co., Milford, MA). Individual lipids were identified by direct infusion in positive ion mode with precursor and neutral loss scans (Xiao et al., 2010; Peters et al., 2010; Li et al., 2014), using the scans shown in **Supplemental Table 4**. Response factor corrections were applied to the MGDG and DGDG analyses to correct for differences in the response of the mass spectrometer to unsaturated galactolipid species as compared to the saturated internal standards. Phospholipid data did not require such response factor corrections, as the biological phospholipids and the internal standard have similar response factors.

The metabolomics data from this study are available at the NIH Common Fund’s National Metabolomics Data Repository (NMDR) website, the Metabolomics Workbench (https://www.metabolomicsworkbench.org) (Sud et al., 2016), where it has been assigned Study ID ST002252. The data can be accessed directly *via* its Project DOI: http://dx.doi.org/10.21228/M8KQ6P.

### Statistical analysis

Lipidomics data were gathered from 44 individual samples that represented three or four replicates per +N/-N treatment for each genotype that was evaluated. Collected data were normalized using the upper-quartile normalization method (Bullard et al., 2010). Transcriptomics data were collected from 41 tissue samples that were gathered in parallel to those used for lipidomics analysis. Due to potential contamination of three root samples by leaves– indicated by assessing the abundance of transcripts encoding for 15 genes coding for photosynthetic functions that would be expected to be expressed in leaves and not in roots [i.e., RUBISCO small subunit genes (At1g67090 and At5g38410), photosystem I subunit genes (e.g., AT1G30380 and AT1G52230)] - the root replicate samples (one of each genotype) were removed from further analysis. The remaining 38 samples that represented between three and four replicates of root and leaf tissues of the three genotypes, grown in either (+N) or (-N) conditions were further analyzed. Initially, transcripts which showed undetectable expression levels across all the samples were removed, which provided abundance data for 26093 transcripts in the root samples, and 26356 transcripts in the leaf samples. The transcriptomics data was analyzed by using the DESeq function in R package DESeq2 (Love et al., 2014). Data was normalized by using the median of ratio method (Anders and Huber, 2010) in DESeq2 package.

Three statistical tests were applied to the transcriptomics datasets:

Test 1) comparison between N-treatments for a given genotype, e.g.,


$$log_{2}\left[GeneExpression_{Genotype1(-N)}\right]–log_{2}\left[GeneExpression_{Genotype1(+N)}\right];$$

Test 2) comparison between genotypes for a given N-condition (+ or -), e.g.,


$$log_{2}\left[Gene\;Expression_{Genotype1(+N)}\right]\;–\;log_{2}\left[Gene\;Expression_{Genotype2(+N)}\right];$$

Test 3) N-treatment effect tests among the 3 genotypes, e.g.,


$$\left(log_{2}\left[Gene\;Expression_{Genotype1(-N)}\right]–\;log_{2}\left[Gene\;Expression_{Genotype1(+N)}\right]\right)$$

$$\;–\;\left(log_{2}\left[Gene\;Expression_{Genotype2(-N)}\right]\;-log_{2}\left[Gene\;Expression_{Genotype2(+N)}\right]\right)$$

Pearson’s correlation was calculated between logcpm, which was defined by log_2_[10^6^• (*r_i,j_* + *δ*)/*R_j_*], where *r_i,j_* is the expression data for transcript *i*, *δ* is the half of the smallest non-zero measurement/count, *R_j_* is the upper quartile for sample *j*. The log fold change (LFC) and q-values (Nettleton et al., 2006) which were calculated from p-values were used for interpretation of the results.

Principal component analysis (PCA) was conducted using the pcomp function of the factoextra package in R. Volcano plots were generated by plotting the negative logarithm of the p value on the y-axis and the LFC on the x-axis in Excel. Heat maps of gene expression levels were created with Perseus (https://maxquant.net/perseus/) (Tyanova et al., 2016).

### Gene ontology analysis

Gene lists were subjected to analysis *via* DAVID (https://david.ncifcrf.gov/home.jsp) (Huang et al., 2009; d. W. Huang and Lempicki, 2009). Function annotation chart was generated by DAVID for each gene list, and terms for Biological Process, Cellular Component and Molecular Function were further reduced using REVIGO (http://revigo.irb.hr/), choosing “Small (0.5)” as the option for reduction (Supek et al., 2011). The fold-enrichment and p-value from DAVID is matched to the reduced lists and plotted.

### MALDI mass spectrometric imaging

Leaves were prepared for mass spectrometric imaging as previously described (Klein et al., 2015). The first pair of true leaves were collected and the abaxial side was adhered to Scotch box sealing tape (3M, Maplewood, MN). After 1-2 h drying in a vacuum, the packing tape was folded so that the adaxial surface of the leaves adhered to the tape, and the tape-encased leaf-tape “sandwich” was passed through a rolling mill to ensure adherence of the tape to the surfaces of the leaf. The two adhering tapes were separated, which resulted in fracturing of the leaf through the mesophyll cell interior, resulting in two separated pieces of packing tape, each retaining the abaxial and adaxial halves of the leaf. Each of these pieces of packing tape, with the exposed leaf surface facing upward, were adhered to a microscope slide using double-sided tape. After acquiring optical images, the samples were subjected to matrix deposition by sublimating (Hankin et al., 2007) 2,5-dihydroxybenzoic acid (DHB; 98%; MilliporeSigma) or 1,5-diaminonaphthalene (DAN; 97%; MilliporeSigma) at 140°C for 4 min at a pressure of~50 mtorr.

Mass spectrometry imaging data were collected using a MALDI-linear ion trap-Orbitrap mass spectrometer (MALDI-LTQ-Orbitrap Discovery; Thermo Fisher Scientific). The instrument was modified to incorporate an external 355 nm frequency tripled Nd : YAG laser (UVFQ; Elforlight, Daventry, United Kingdom) and a f=60 mm focus lens (Feenstra et al., 2017). TunePlus and XCalibur (Thermo Fisher Scientific) were used to define imaging parameters and to acquire data, respectively. Leaves were acquired using 100 μm raster step size. Mass spectra were acquired with 10 laser shots per spectrum mostly using an Orbitrap mass analyzer (resolution of 30,000 at *m/z* 400) for an *m/z* scan range of 200–1000. Data were obtained in positive and negative ion mode, using DHB and DAN, respectively.

MS images were generated using ImageQuest (Thermo Fisher Scientific) with a mass window of ±5 ppm, and without normalization. The same minimum and maximum plot values were used across all images and compounds. Peak assignments were based on accurate mass measurement, and comparison with online databases (METLIN, https://metlin.scripps.edu). Images were saved from a screenshot of the 2-D image window.

## Data availability statement

The datasets presented in this study can be found in online repositories. The names of the repository/repositories and accession number(s) can be found below: https://www.ncbi.nlm.nih.gov/, PRJNA769614 http://dx.doi.org/10.21228/M8KQ6P,ST002252.

## Author contributions

BN, DB contributed to conception and design of the study. GD, YM, MD, LL gathered the data, organized the data. ML, DN, XZ performed the statistical analysis. BN, DB, YJL, DN provided mentorship and project administration. GD, YM, MD, wrote sections of the manuscript. All authors contributed to the article and approved the submitted version. 

## Supplementary material

Supplementary Figure 1 Experimental workflow for tissue preparation. Schematic representation of the workflow used to prepare root and leaf tissue samples from seedlings grown in (+N) and (-N) conditions.

Supplementary Figure 2 GO enrichment analysis of genes that are differentially expressed in the *atg7* and *atg9* mutant. GO enrichment terms that are common to leaves and roots and identified from downregulated and upregulated DEGs in the comparison between *atg7* and WT, or *atg9* and WT, in (-N)-conditions.

Supplementary Figure 3 Identification of genes that show differential expression in response to the alteration in the autophagic state of the tissue or nitrogen status. **(A)** The graph illustrates the expression response of a hypothetical gene as the ratio of expression in (-N) versus (+N) conditions that induce autophagy in either WT plants (green datapoints) or mutant plants (either *atg7* or *atg9*) (orange datapoints). Genes that show expression patterns where the green and orange datapoints are statistically distinguishable from each other we define as genes that are responding to autophagy, whereas genes that show expression patterns where the green and orange datapoints are statistically indistinguishably separated are responding to the (-N)-treatment. The numbers of the DEGs associated with each expression pattern identified in leaves and roots of the *atg7* or *atg9* mutants are tabularized next to the illustrative graph. **(B)** Venn diagram visualization of the number of DEGs responding autophagy in the leaves and roots of the *atg7* or *atg9* mutants. **(C)** Venn diagram visualization of the number of DEGs responding to the (-N)-treatment and not to autophagy, in the leaves and roots of the *atg7* or *atg9* mutants.

Supplementary Figure 4 Biological process GO terms of the down- and up-regulated genes in (-N) condition that are associated with nitrogen status in the *atg7* vs. WT and *atg9* vs. WT comparisons. The statistical significance of the enrichment of each term, evaluated by log_10_ p-value, is indicated by the intensity of the blue color shading the fold-enrichment data.

Supplementary Figure 5 Cellular component GO terms of the down- and up-regulated genes in (-N) condition that are associated with nitrogen status in the *atg7* vs. WT and *atg9* vs. WT comparisons. The statistical significance of the enrichment of each term, evaluated by log_10_ p-value, is indicated by the intensity of the blue color shading the fold-enrichment data.

Supplementary Figure 6 Molecular function GO terms of the down- and up-regulated genes in (-N) condition that are associated with nitrogen status in the *atg7* vs. WT and *atg9* vs. WT comparisons. The statistical significance of the enrichment of each term, evaluated by log_10_ p-value, is indicated by the intensity of the blue color shading the fold-enrichment data.

Supplementary Figure 7 GO analysis of genes that are differentially expressed upon alterations in autophagy (all levels of enrichment).

Supplementary Figure 8 Volcano plot representation of the lipidome, comparing the relative abundance of individual lipids between WT and *atg9* leaf grown in (+N) condition **(A)**; between WT and *atg9* leaf grown in -N conditions **(B)**; between WT and *atg9* roots grown in (+N) condition **(C)**; and between WT and *atg9* roots grown in (-N).

Supplementary Figure 9 Evaluation of the reproducibility of mass spectrometric imaging data. The optical images and MS images of the spatial distribution of the deprotonated ion of lysoPG(18:2)-H, which was acquired in negative mode using DAN as the matrix. Images were acquired from three replicate leaves, each from a different plant, of the indicated genotype, that was grown in either in (+N)- or (-N)-conditions. Leaves were fractured longitudinally to expose the adaxial and abaxial surfaces of the leaf interior, which were imaged by mass spectrometry. The ion signals were normalized to the total ion count and the maximum values used was 1x10^5^.
