# Supplementary figures and images for: Biological insights from multi-omics analysis strategies: Complex pleotropic effects associated with autophagy
Source: Front Plant Sci. 2023 Feb 16;14:1093358. doi: 10.3389/fpls.2023.1093358 (PMC9978356; doi:10.3389/fpls.2023.1093358)

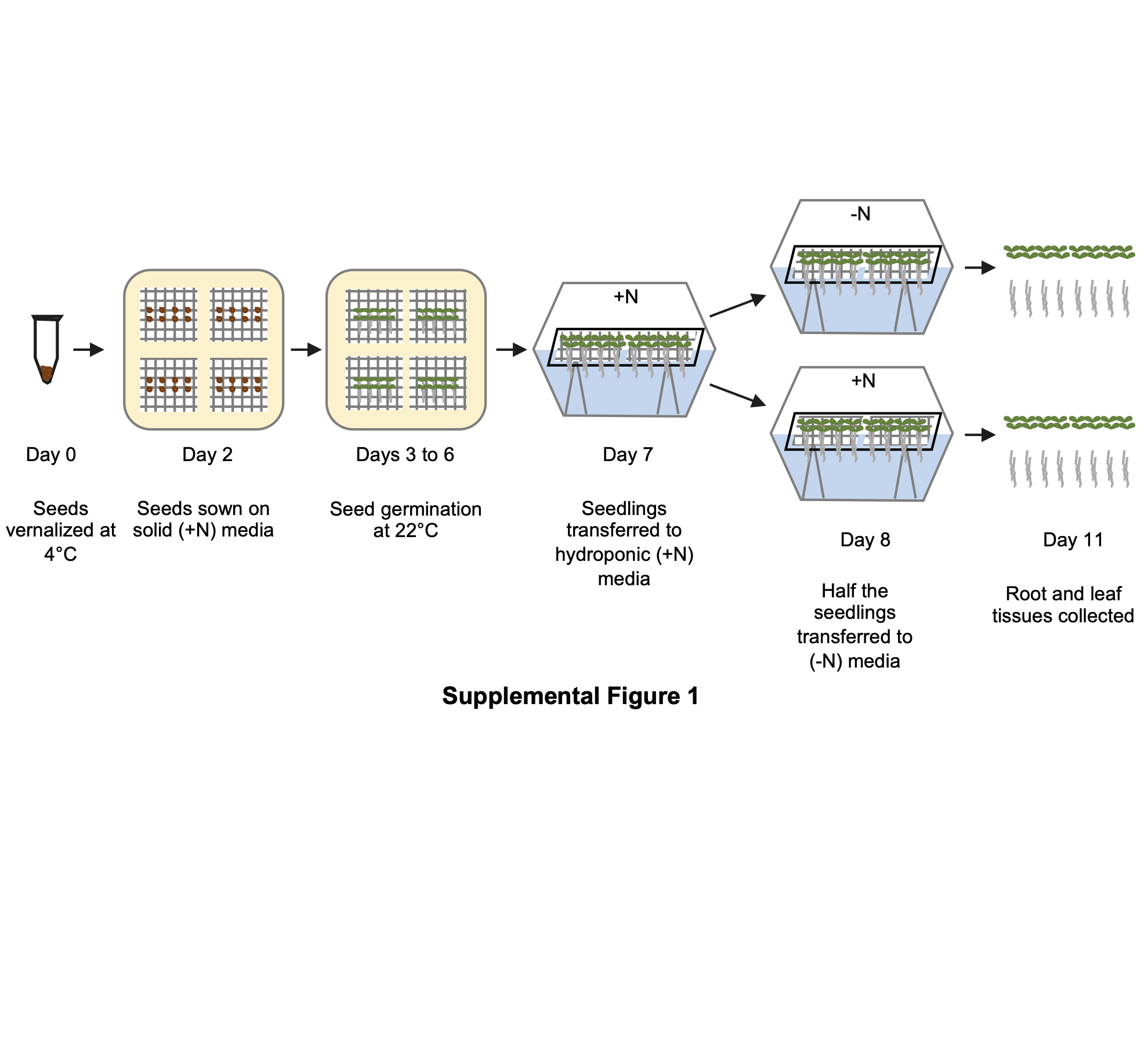

Supplement: Supplementary Figure 1 — Experimental workflow for tissue preparation. Schematic representation of the workflow used to prepare root and leaf tissue samples from seedlings grown in (+N) and (-N) conditions. [file Image_1.tif]

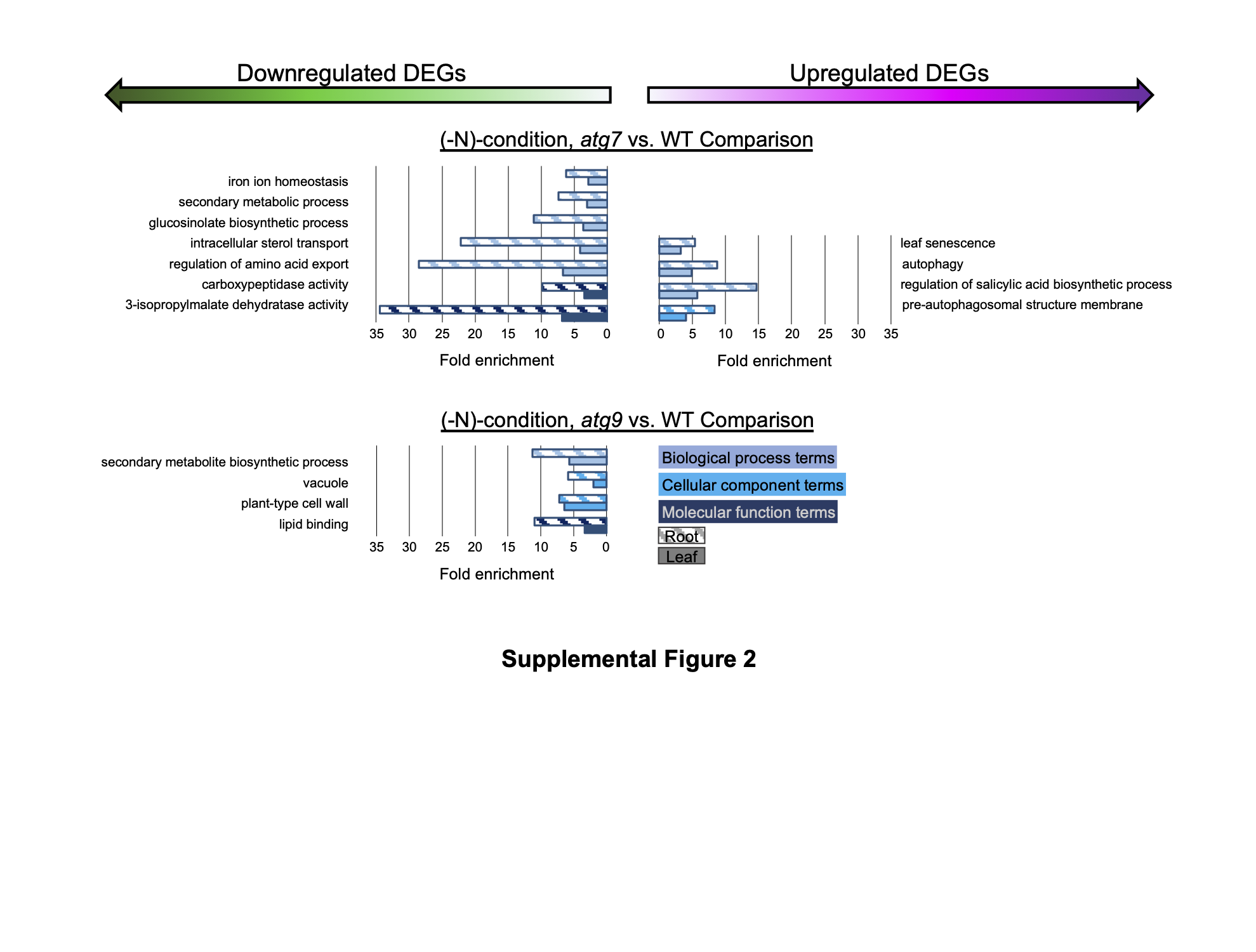

Supplement: Supplementary Figure 2 — GO enrichment analysis of genes that are differentially expressed in the atg7 and atg9 mutant. GO enrichment terms that are common to leaves and roots and identified from downregulated and upregulated DEGs in the comparison between atg7 and WT, or atg9 and WT, in (-N)-conditions. [file Image_2.tif]

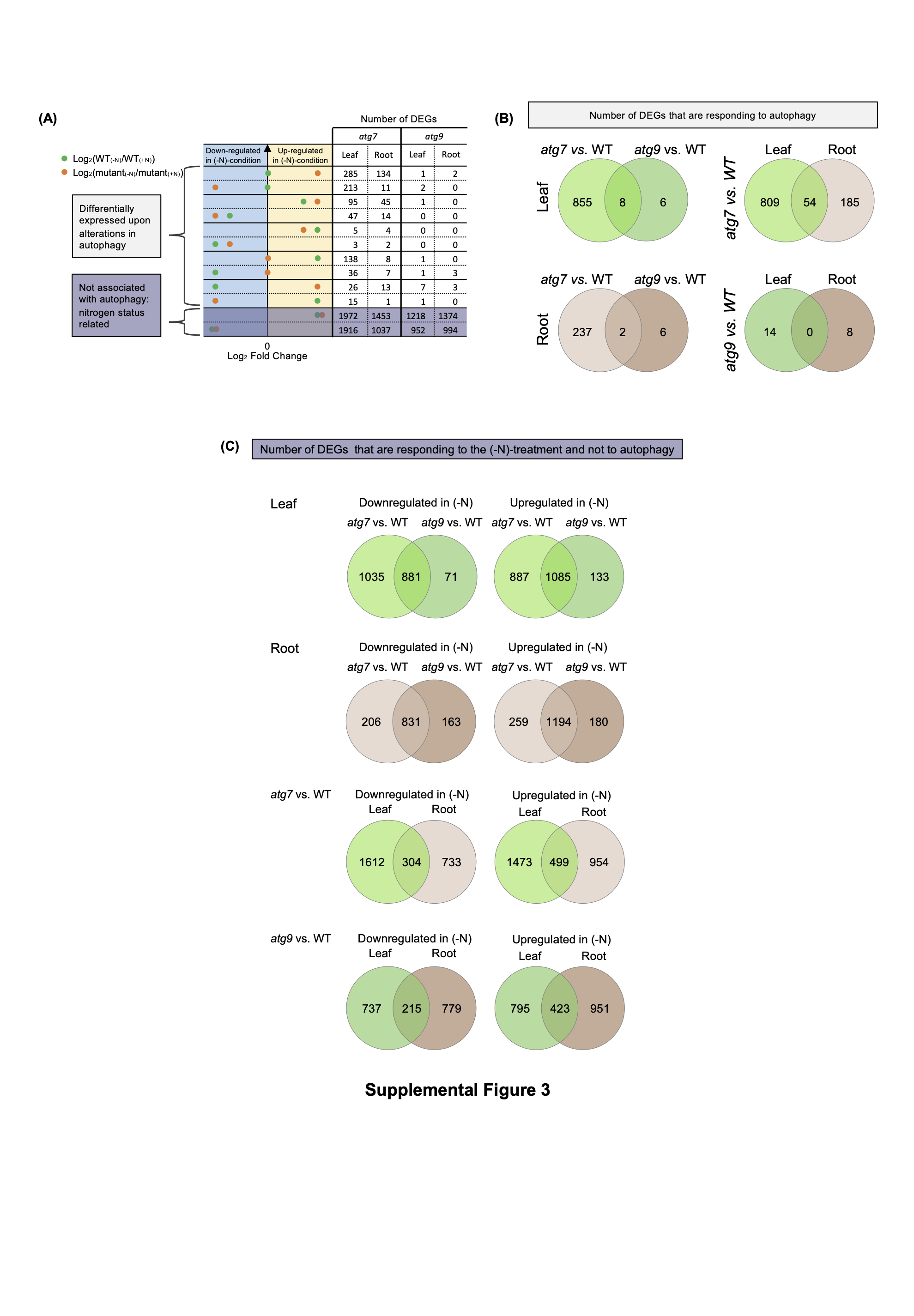

Supplement: Supplementary Figure 3 — Identification of genes that show differential expression in response to the alteration in the autophagic state of the tissue or nitrogen status. (A) The graph illustrates the expression response of a hypothetical gene as the ratio of expression in (-N) versus (+N) conditions that induce autophagy in either WT plants (green datapoints) or mutant plants (either atg7 or atg9) (orange datapoints). Genes that show expression patterns where the green and orange datapoints are statistically distinguishable from each other we define as genes that are responding to autophagy, whereas genes that show expression patterns where the green and orange datapoints are statistically indistinguishably separated are responding to the (-N)-treatment. The numbers of the DEGs associated with each expression pattern identified in leaves and roots of the atg7 or atg9 mutants are tabularized next to the illustrative graph. (B) Venn diagram visualization of the number of DEGs responding autophagy in the leaves and roots of the atg7 or atg9 mutants. (C) Venn diagram visualization of the number of DEGs responding to the (-N)-treatment and not to autophagy, in the leaves and roots of the atg7 or atg9 mutants. [file Image_3.tif]

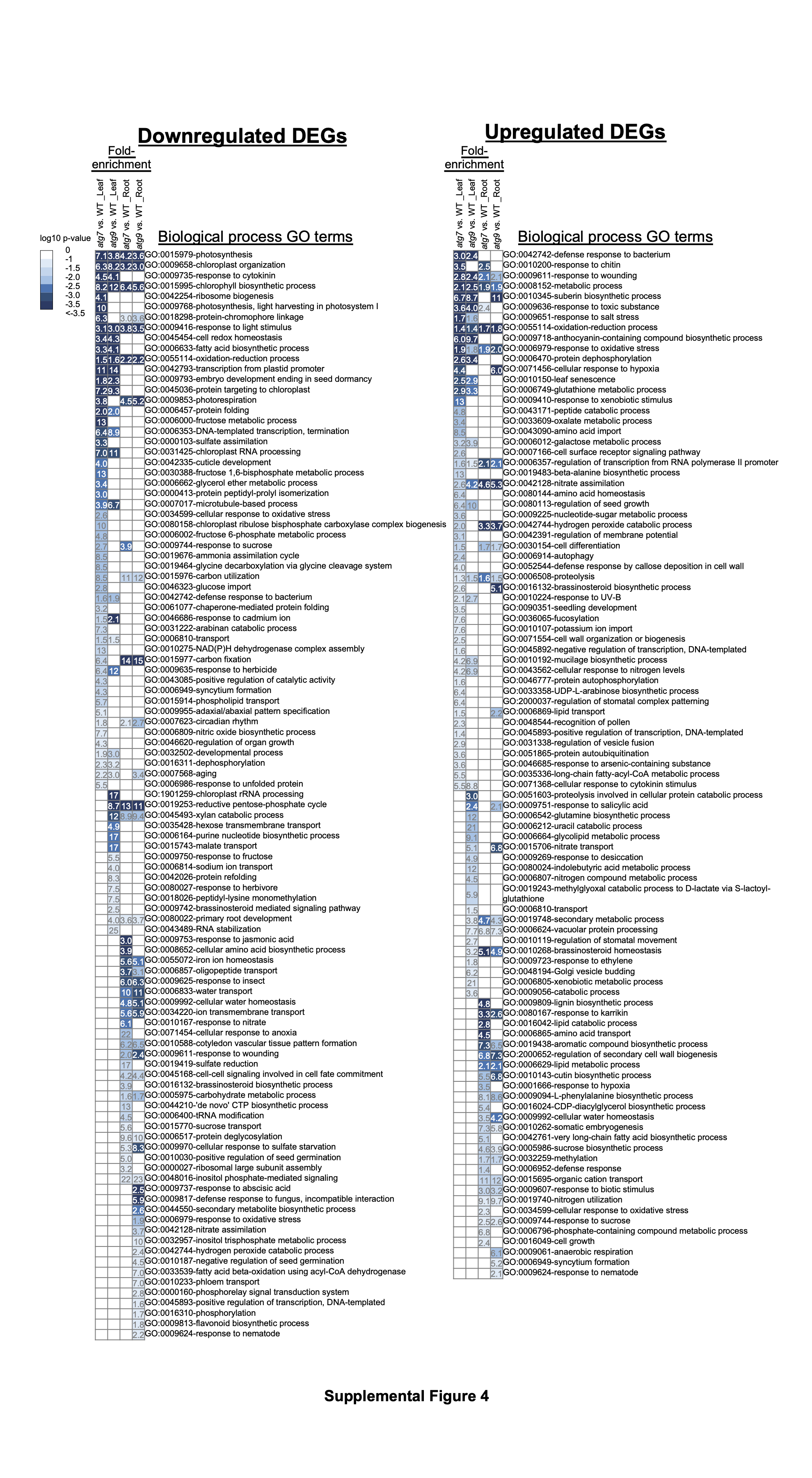

Supplement: Supplementary Figure 4 — Biological process GO terms of the down- and up-regulated genes in (-N) condition that are associated with nitrogen status in the atg7 vs. WT and atg9 vs. WT comparisons. The statistical significance of the enrichment of each term, evaluated by log10 p-value, is indicated by the intensity of the blue color shading the fold-enrichment data. [file Image_4.tif]

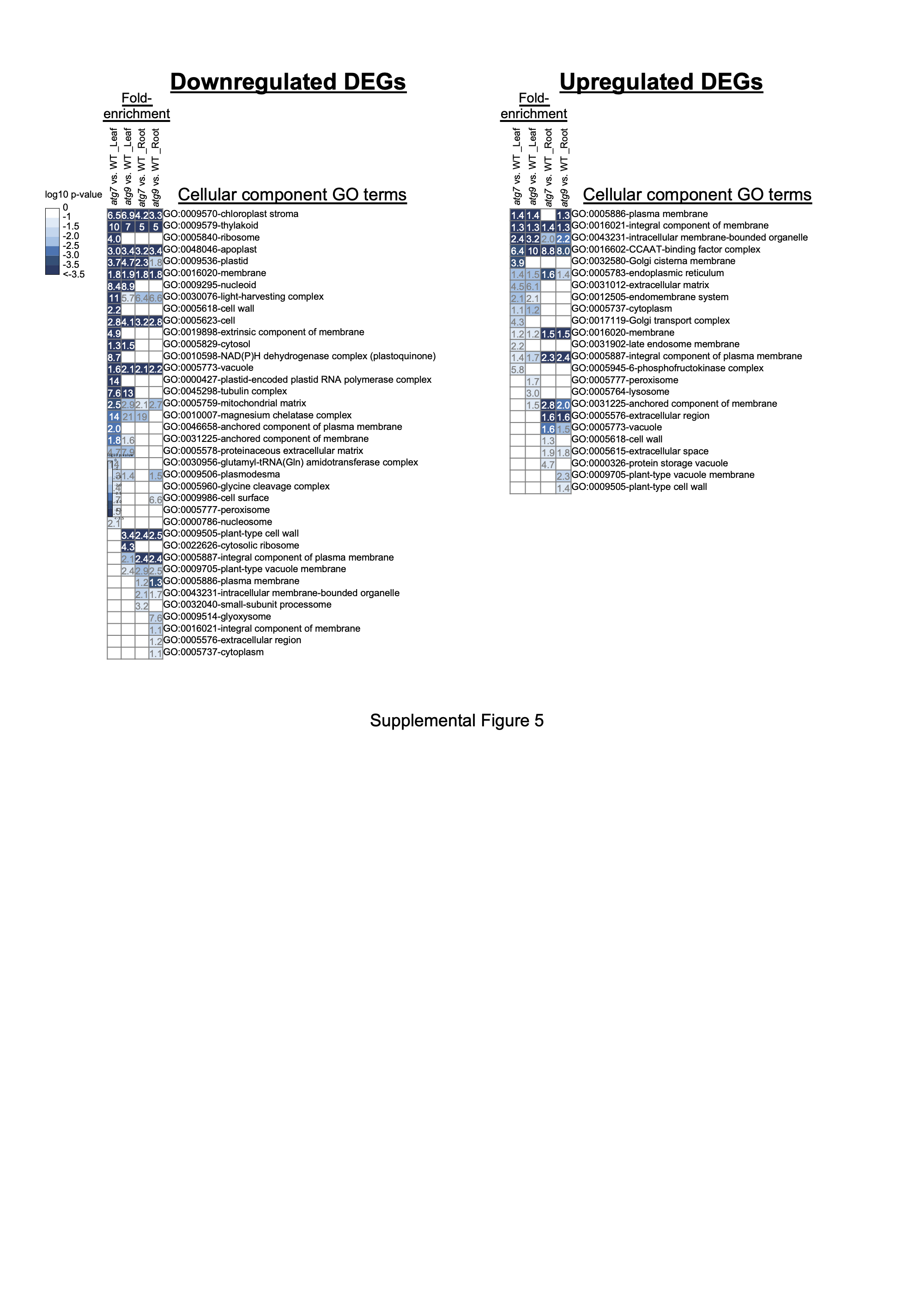

Supplement: Supplementary Figure 5 — Cellular component GO terms of the down- and up-regulated genes in (-N) condition that are associated with nitrogen status in the atg7 vs. WT and atg9 vs. WT comparisons. The statistical significance of the enrichment of each term, evaluated by log10 p-value, is indicated by the intensity of the blue color shading the fold-enrichment data. [file Image_5.tif]

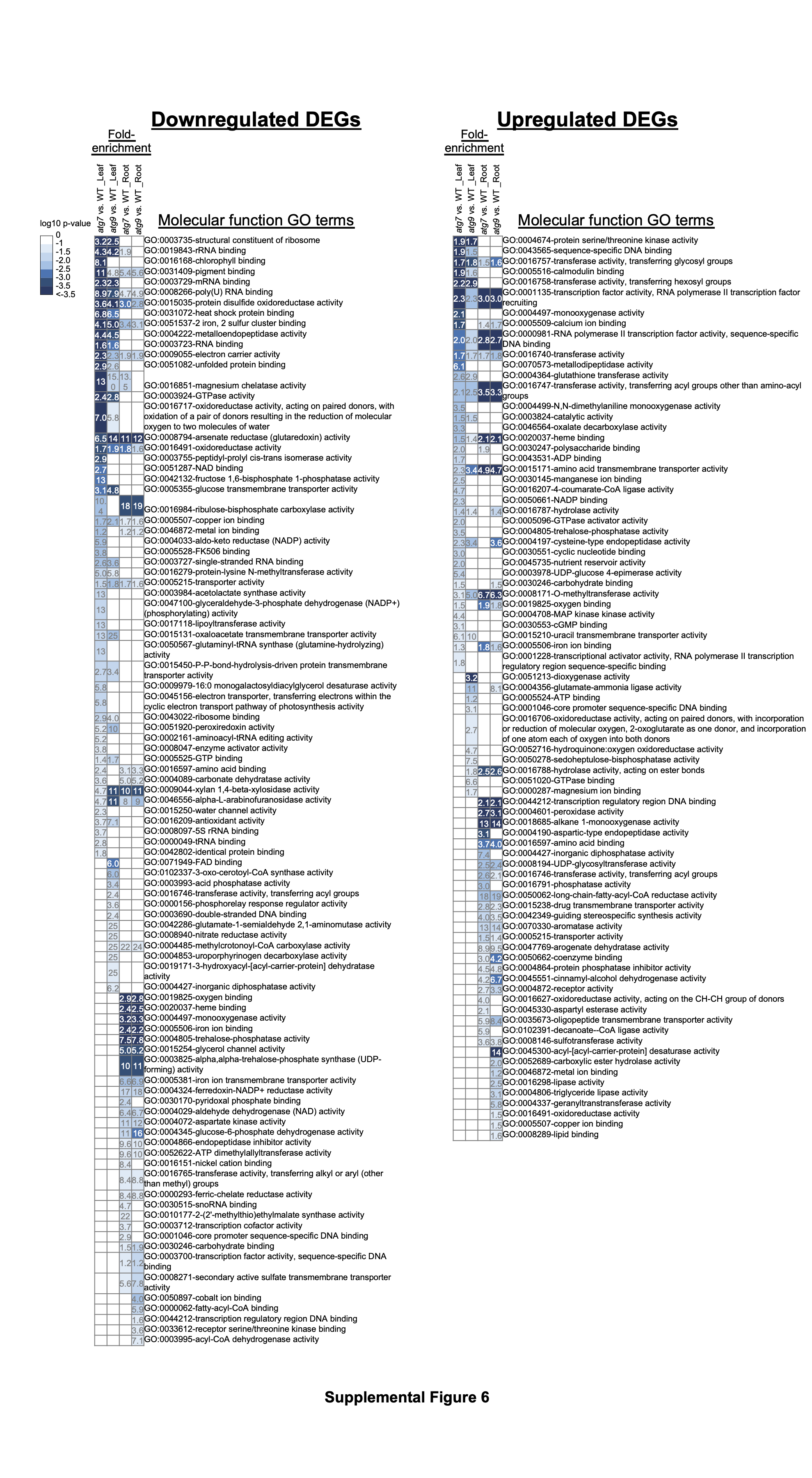

Supplement: Supplementary Figure 6 — Molecular function GO terms of the down- and up-regulated genes in (-N) condition that are associated with nitrogen status in the atg7 vs. WT and atg9 vs. WT comparisons. The statistical significance of the enrichment of each term, evaluated by log10 p-value, is indicated by the intensity of the blue color shading the fold-enrichment data. [file Image_6.tif]

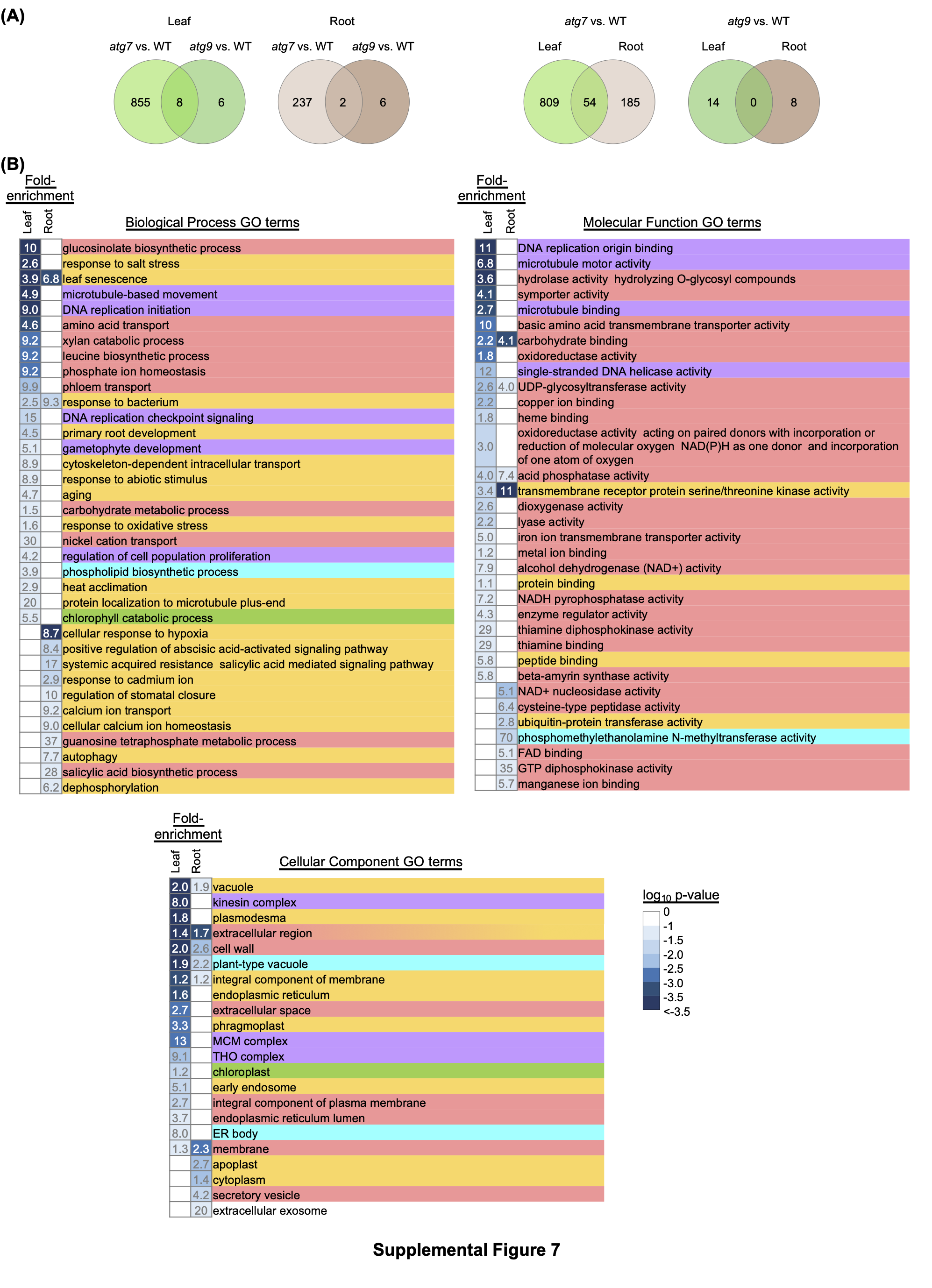

Supplement: Supplementary Figure 7 — GO analysis of genes that are differentially expressed upon alterations in autophagy (all levels of enrichment). [file Image_7.tif]

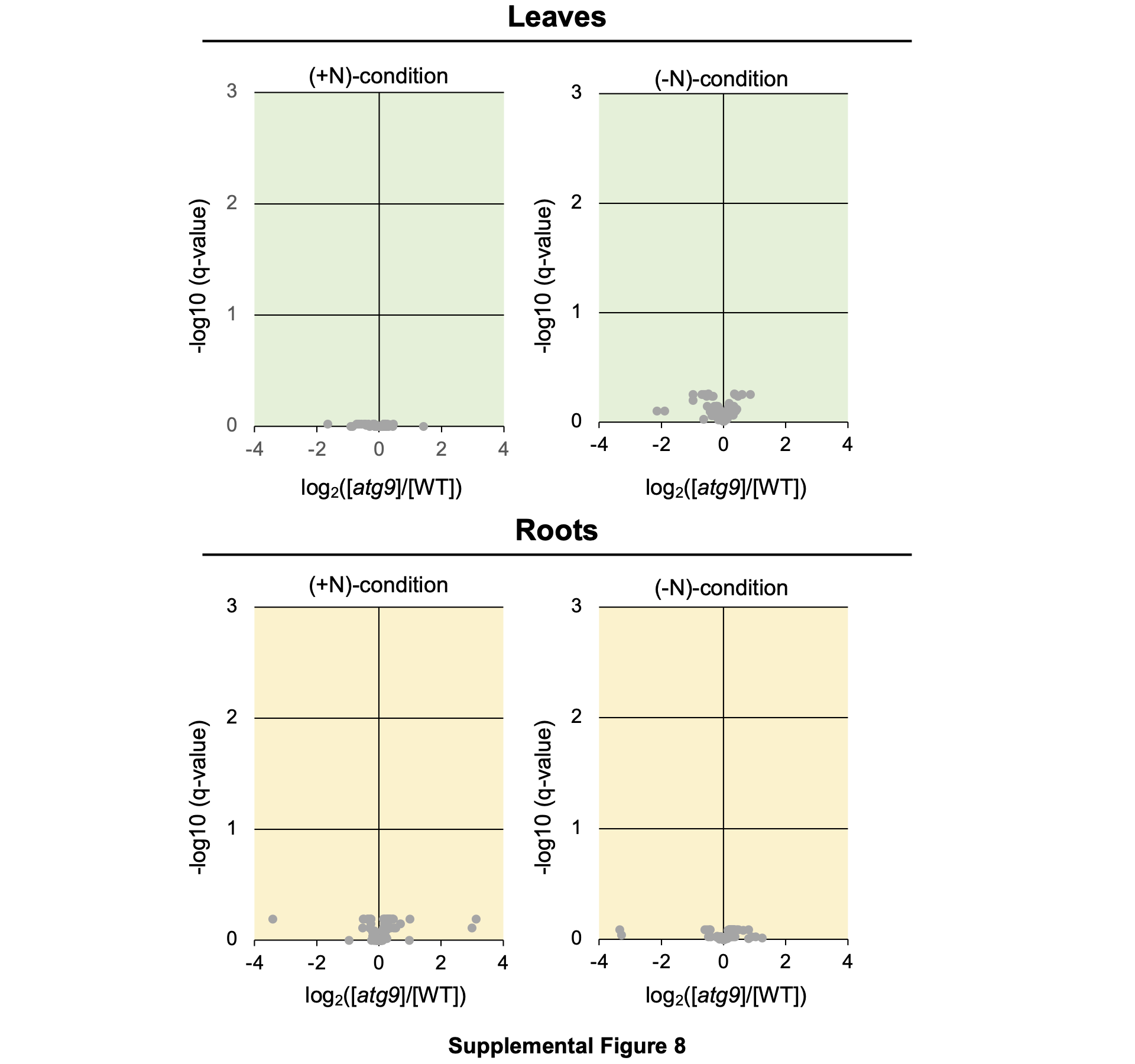

Supplement: Supplementary Figure 8 — Volcano plot representation of the lipidome, comparing the relative abundance of individual lipids between WT and atg9 leaf grown in (+N) condition (A); between WT and atg9 leaf grown in -N conditions (B); between WT and atg9 roots grown in (+N) condition (C); and between WT and atg9 roots grown in (-N). [file Image_8.tif]

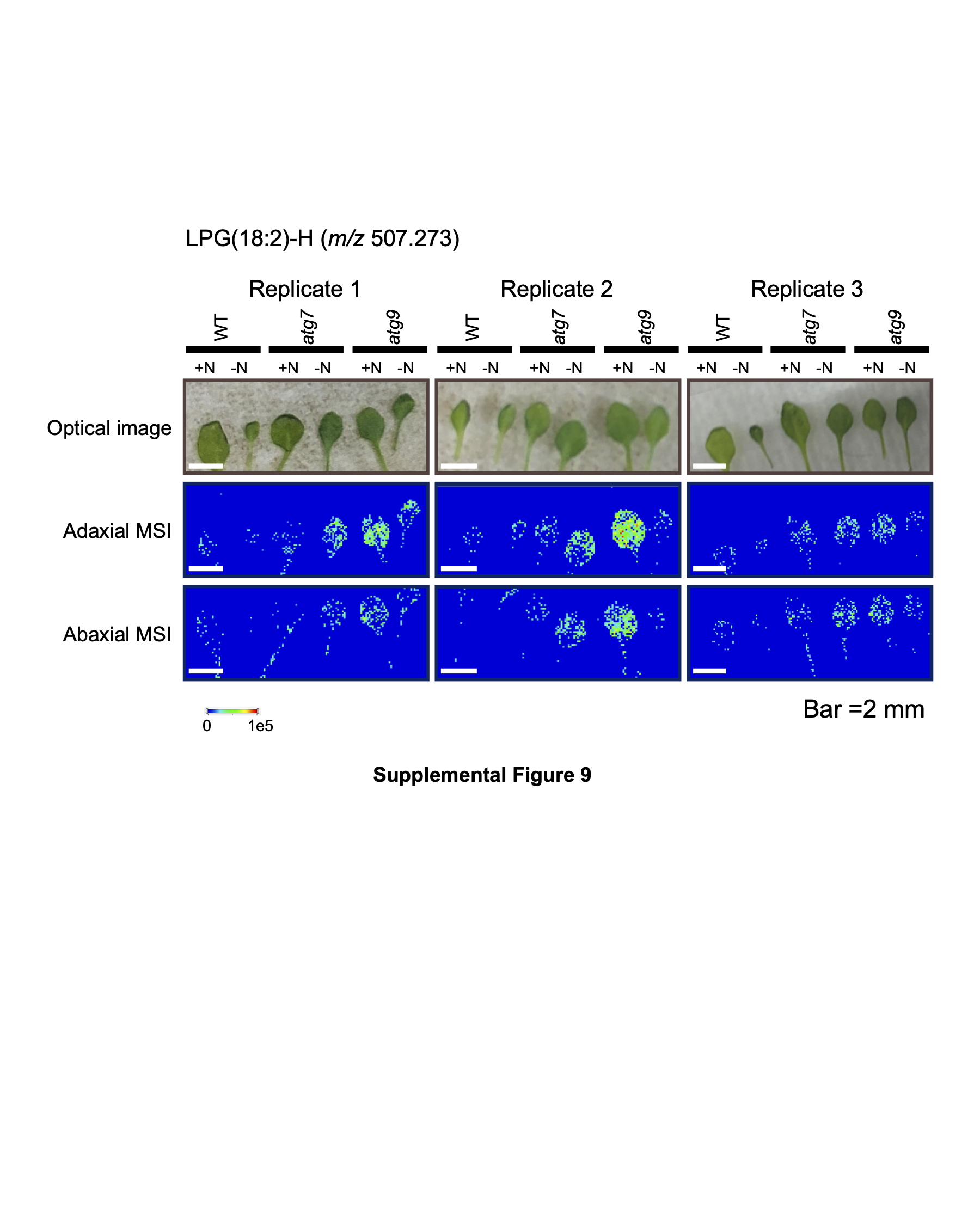

Supplement: Supplementary Figure 9 — Evaluation of the reproducibility of mass spectrometric imaging data. The optical images and MS images of the spatial distribution of the deprotonated ion of lysoPG(18:2)-H, which was acquired in negative mode using DAN as the matrix. Images were acquired from three replicate leaves, each from a different plant, of the indicated genotype, that was grown in either in (+N)- or (-N)-conditions. Leaves were fractured longitudinally to expose the adaxial and abaxial surfaces of the leaf interior, which were imaged by mass spectrometry. The ion signals were normalized to the total ion count and the maximum values used was 1x10^5^. [file Image_9.tif]

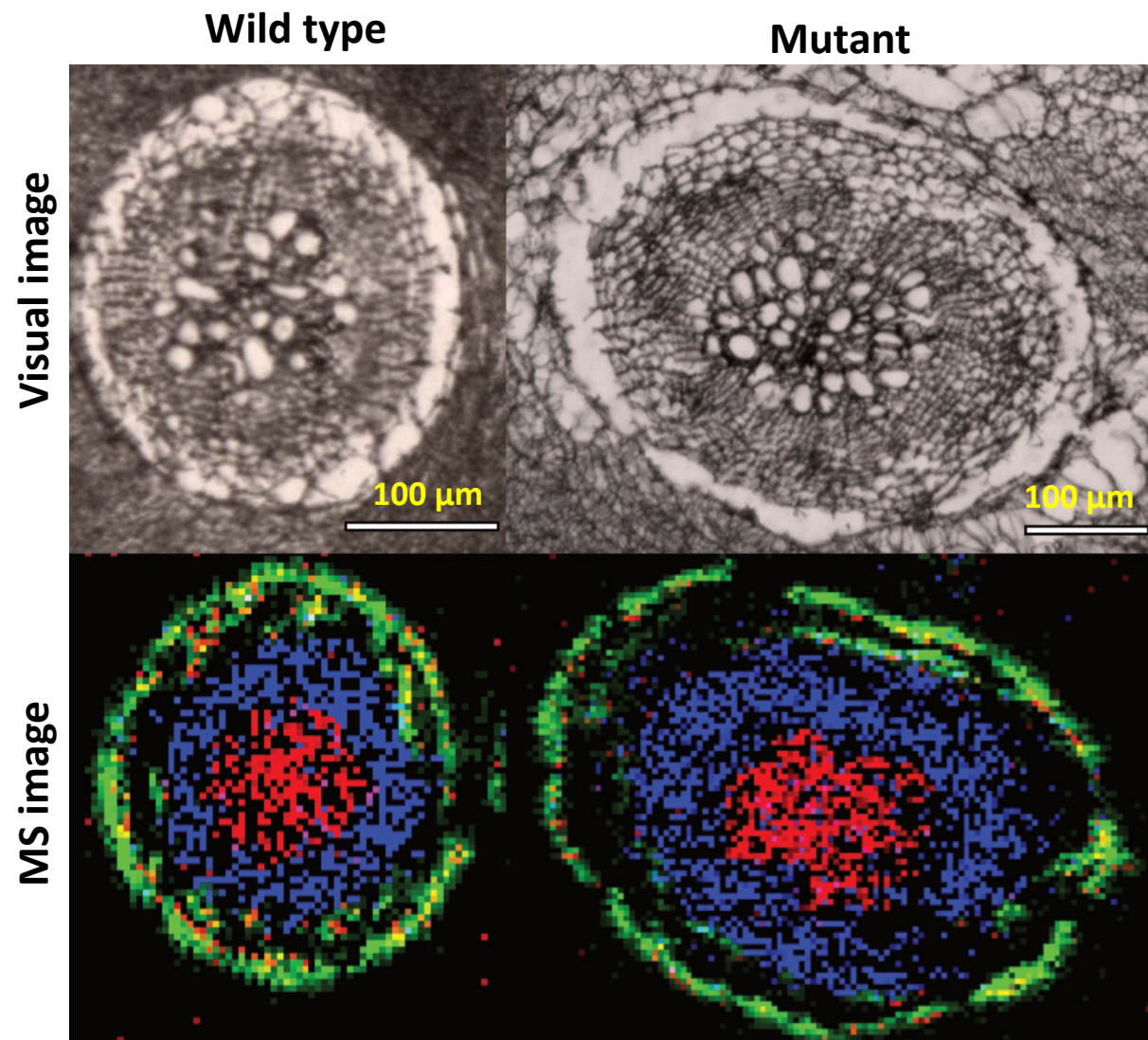

Metabolites detected by MSI

Blue: PI(34:2) ( $m/z$  833.516)

Red: Unknown ( $m/z$  267.066)

Green: Unknown ( $m/z$  459.347)

Supplemental Figure 10

Supplement: Supplementary file 10 [file Image_10.pdf]
