# Supplementary material for: Biological insights from multi-omics analysis strategies: Complex pleotropic effects associated with autophagy
Source: Front Plant Sci. 2023 Feb 16;14:1093358. doi: 10.3389/fpls.2023.1093358 (PMC9978356; doi:10.3389/fpls.2023.1093358)
Supplement: Supplementary file 11 [file Table_1.pdf]

Supplemental Table 1. Number of differentially expressed genes in leaves and roots of WT, and *atg7* and *atg9* mutants grown in normal (+N) or autophagy-inducing (-N) conditions (statistical significance level of q<0.05)[a]

		Leaf				Root			
		Up-regulated		Down-regulated		Up-regulated		Down-regulated	
	Comparison	Genome-wide[b]	Lipid genes[c]	Genome-wide	Lipid genes	Genome-wide	Lipid genes	Genome-wide	Lipid genes
1	WT(-N)/WT(+N)	3035	107	2385	97	1544	100	1188	24
2	*atg7*(-N)/*atg7*(+N)	3700	144	3284	100	2666	156	1710	31
3	*atg9*(-N)/*atg9*(+N)	1434	62	953	37	1776	101	1123	21
4	*atg7*/WT, (+N)	14	0	3	0	57	1	91	2
5	*atg7*/WT, (-N)	1719	73	2013	36	573	35	243	5
6	*atg9*/WT, (+N)	11	0	19	1	16	1	27	0
7	*atg9*/WT, (-N)	45	3	117	4	24	0	30	4

[a] List of differentially expressed genes are tabulated in Supplemental Table 2.
[b] Among all annotated genes in the Arabidopsis genome that show statistically significant differential expression (q<0.05).
[b] Among the lipid metabolism associated genes identified in the AraLip database (http://aralip.plantbiology.msu.edu) that show statistically significant differential expression (q<0.05)
