# Supplementary material for: Biological insights from multi-omics analysis strategies: Complex pleotropic effects associated with autophagy
Source: Front Plant Sci. 2023 Feb 16;14:1093358. doi: 10.3389/fpls.2023.1093358 (PMC9978356; doi:10.3389/fpls.2023.1093358)
Supplement: Supplementary file 14 [file Table_4.docx]

| Supplemental Table 4. Mass spectrometry parameters for lipidomics analysis on Waters Xevo TQS (electrospray ionization triple quadrupole mass spectrometer). | | | | | | | | | | | |
| --- | --- | --- | --- | --- | --- | --- | --- | --- | --- | --- | --- |
| **Lipid class** | **Adduct identified** | | **Scan mode^a^** | | **Cone voltage (V)** | | **Collision energy (V)** | | **Internal standards^b^** | |  |
| DGDG | [M + NH_4_]^+^ | | NL 341.11 | | 40 | | 16 | | DGDG(34:0); DGDG(36:0) | |  |
| LPC, PC | [M + H]+ | | Pre 184.07 | | 40 | | 28 | | LPC(13:0); LPC(19:0) | |  |
| LPE, PE | [M + H]+ | | NL 141.02 | | 40 | | 12 | | LPE(14:0); LPE(18:0) | |  |
| LPG | [M + NH_4_]^+^ | | NL 189.04 | | 25 | | 10 | | LPG(14:0); LPG(18:0) | |  |
| MGDG | [M + NH_4_]^+^ | | NL 179.06 | | 40 | | 14 | | MGDG(34:0); MGDG(36:0) | |  |
| PA | [M + NH_4_]^+^ | | NL 115.00 | | 40 | | 5 | | PA(28:0); PA(40:0) | |  |
| PG | [M + NH_4_]^+^ | | NL 189.00 | | 40 | | 8 | | PG(28:0); PG(40:0) | |  |
| PI | [M + NH_4_]^+^ | | NL 277.00 | | 40 | | 17 | | PI(34:0); PI(36:0) | |  |
| PS | [M + H]+ | | NL 185.00 | | 40 | | 13 | | PS(28:0); PS(40:0) | |  |
|  | |  | |  | |  | |  | |  | |
|  | |  | |  | |  | |  | |  | |

^a^Scan modes: NL, neutral loss; Pre, Precursor.

^b^Known amounts of the two internal standards were added to the samples. Quantification of biological analytes was performed by comparing the spectral intensity of the biological analytes to the spectral intensity/molar amount of both standards as a function of *m/z.*
